# Targeting FTL regulates ferroptosis and remodels lymph node metastasis microenvironment in esophageal squamous cell carcinoma

**DOI:** 10.7150/ijbs.112017

**Published:** 2025-10-16

**Authors:** Shuyue Zheng, Yunzhi Liu, Baifeng Zhang, Jiao Huang, Xiaona Fang, Cuicui Huang, Lanqi Gong, Jie Luo, Yuma Yang, Shan Liu, Ching Ngar Wong, Jinlin Huang, Shanshan Li, Yanan Tan, Qingyun Chen, Yanru Qin, Xin-Yuan Guan

**Affiliations:** 1 Department of Clinical Oncology, The University of Hong Kong-Shenzhen Hospital, Shenzhen, China.; 2 Department of Clinical Oncology, Li Ka Shing Faculty of Medicine, The University of Hong Kong, Hong Kong, SAR China.; 3 State Key Laboratory of Liver Research, Li Ka Shing Faculty of Medicine, The University of Hong Kong, Hong Kong, SAR China.; 4 State Key Laboratory of Oncology in Southern China, Sun Yat-sen University Cancer Center, Guangzhou, China.; 5 Department of Clinical Oncology, The First Affiliated Hospital, Zhengzhou University, Zhengzhou, China.; 6 Advanced Nuclear Energy and Nuclear Technology Research Center, Advanced Energy Science and Technology Guangdong Laboratory, Huizhou, China.; 7 MOE Key Laboratory of Tumor Molecular Biology, Jinan University, Guangzhou, China.; 8 Department of Pediatric Oncology, Sun Yat-sen University Cancer Center, Guangzhou, China.; 9 Medical Research Institute, Guangdong Provincial People's Hospital, Southern Medical University, Guangzhou, Guangdong, 510080, China.; 10 Shanghai Clinical Research and Trial Center, Shanghai, China, China.; 11 ShanghaiTech University, Shanghai, China.

**Keywords:** ferroptosis, metastasis, tumor microenvironment, esophageal squamous carcinoma

## Abstract

More than half of Esophageal squamous cell carcinoma (ESCC) patients are at an advanced stage when first diagnosed, thus they do not benefit much from radical surgery. Single-cell RNA sequencing (scRNA-seq) data from patients with ESCC lymph node metastasis in our laboratory implied that ferroptosis might play an important role in ESCC metastasis. Ferroptosis was found to be a shared specific pathway between ESCC and adjacent non-tumor tissue, as well as between ESCC lymph node metastasis and adjacent non-tumor tissue, of which FTL was selected as the pivotal target gene within this common pathway. Bioinformatic analyses showed that FTL was highly expressed in both primary and metastatic sites than normal, and patients with high expression had poor prognosis, and its function was related to macrophages in TME. Functional studies have shown that FTL promoted tumor growth, tolerated oxidative stress, reduced the sensitivity of ESCC cells to ferroptosis, facilitated epithelial-mesenchymal transition (EMT) and recruited more macrophages to promote metastasis. Mechanism studies have shown that FTL promotes ESCC development and metastasis via NRF2 pathway and inhibits ferroptosis via NCOA4 protein. *In vivo* treatment, Brusatol, was found to inhibit FTL expression and have a significant inhibitory effect on ESCC growth and metastasis.

## Introduction

Esophageal squamous cell carcinoma (ESCC) has a high incidence and mortality and is one of the most common cancers in the world^1^. Radical surgery for esophageal cancer is currently the only mainstream clinical method to cure esophageal squamous cell carcinoma and make a complete recovery for ESCC patients, however, more than half of esophageal squamous cell carcinoma patients are at an advanced stage when first diagnosed^2^. Such patients could not undergo the surgery due to extensive metastasis. Several novel immune and targeted drugs for advanced ESCC therapy have been approved by the U.S. Food and Drug Administration (FDA), such as programmed cell death protein 1 (PD-1) inhibitors and human epidermal growth factor receptor 2 (Her-2) inhibitors, but the survival rate in advanced patients remain low^3^, ^4^. The 5-year survival rate for advanced esophageal cancer is reported to be only 19%, and local invasion and distant metastasis of ESCC are the main causes of treatment failure in advanced patients^5^, ^6^. Therefore, further molecular studies of the ESCC landscape have the potential to identify new biomarkers and molecular targets that influence the progression of ESCC and enable the design of new therapeutic strategies based on this^7^.

Recent studies showed that the plasticity of tumor cells enabled metabolic reprogramming to recruit and modify cells and make them more susceptible to metastasis^8^, ^9^. This metabolic reprogramming is related to the sensitivity of tumor cells to ferroptosis^10^. Therefore, finding suitable ferroptosis targets to improve the sensitivity of cancer cells to ferroptosis via carcinogenic pathways and metabolic reprogramming is critical for the development of new targeted drugs for ESCC and its metastasis. Single-cell RNA sequencing (scRNA-seq) technology represents a groundbreaking advancement in genomic research, enabling unprecedented resolution in cellular analysis. Unlike bulk sequencing that averages signals across cell populations, this technique profiles individual cells to uncover hidden heterogeneity within tissues. Key advantages include the ability to identify rare cell subtypes, trace developmental trajectories, and detect subtle transcriptional variations masked in ensemble data^11^. Therefore, it is currently one of the hottest technologies in the field of cancer research, which can accurately analyze the tumor microenvironment (TME) of the primary tumor and its metastatic tumor from a single-cell perspective^12^. Thus, combining scRNA-seq in ESCC metastasis research provides an excellent opportunity to develop new biomarkers and molecular targets. Our lab has profiled the transcriptome of single cells from four ESCC patients with lymph node metastases and TME map of the entire ESCC metastasis^13^. Through in-depth analysis of this scRNA-seq data, we found that ferroptosis was a shared specific pathway between ESCC and adjacent non-tumor tissue, as well as between ESCC lymph node metastasis and adjacent non-tumor tissue. Intersections of genes from epithelial cells among all groups were taken and FTL was selected as the pivotal target gene within ferroptosis pathway. Thus, we think targeting FTL might be a specific gene to regulate the remodeling of ferroptosis and lymph node microenvironment in ESCC.

Ferritin is the main iron storage protein in prokaryotes and eukaryotes. It is composed of 24 subunits from heavy and light ferritin chain. Ferritin light chain (FTL) is a gene encoding the light subunit of ferritin^14^, ^15^. Changes in the composition of ferritin subunits affect the rate of iron absorption and release in different tissues^16^, ^17^. In recent years, studies have found that FTL is closely related to the occurrence and progression in many cancers except for ESCC. It has been shown that FTL expression was increased in glioblastoma and acute myeloid leukemia, and silencing FTL inhibits glioblastoma cell proliferation through the GADD45/JNK pathway^18^. Also, it has been found that FTL competed with long non-coding RNA to regulate chemotherapy resistance and metastasis in colorectal cancer, and hypoxia induced FTL to promote epithelial mesenchymal transformation (EMT) and chemotherapy resistance in glioma^19^, ^20^. However, at present, there are no studies of targeting FTL in ESCC progression. Our study filled the gap in this part, and proved that FTL might be a promising treatment for the development and metastasis of ESCC.

## Materials and Methods

### Clinical specimens

ESCC tissue microarray (TMA) was used as described previously^21^,^22^. These patients underwent preoperative treatments and signed informed consent forms. All of the ESCC samples with signed informed consent forms were confirmed by the Committees for Ethical Review of Research and the Institutional Review Boards of Sun Yat-sen University Cancer Center (SYSUCC).

### Single cell sequencing of specimens

**Figure 1:**
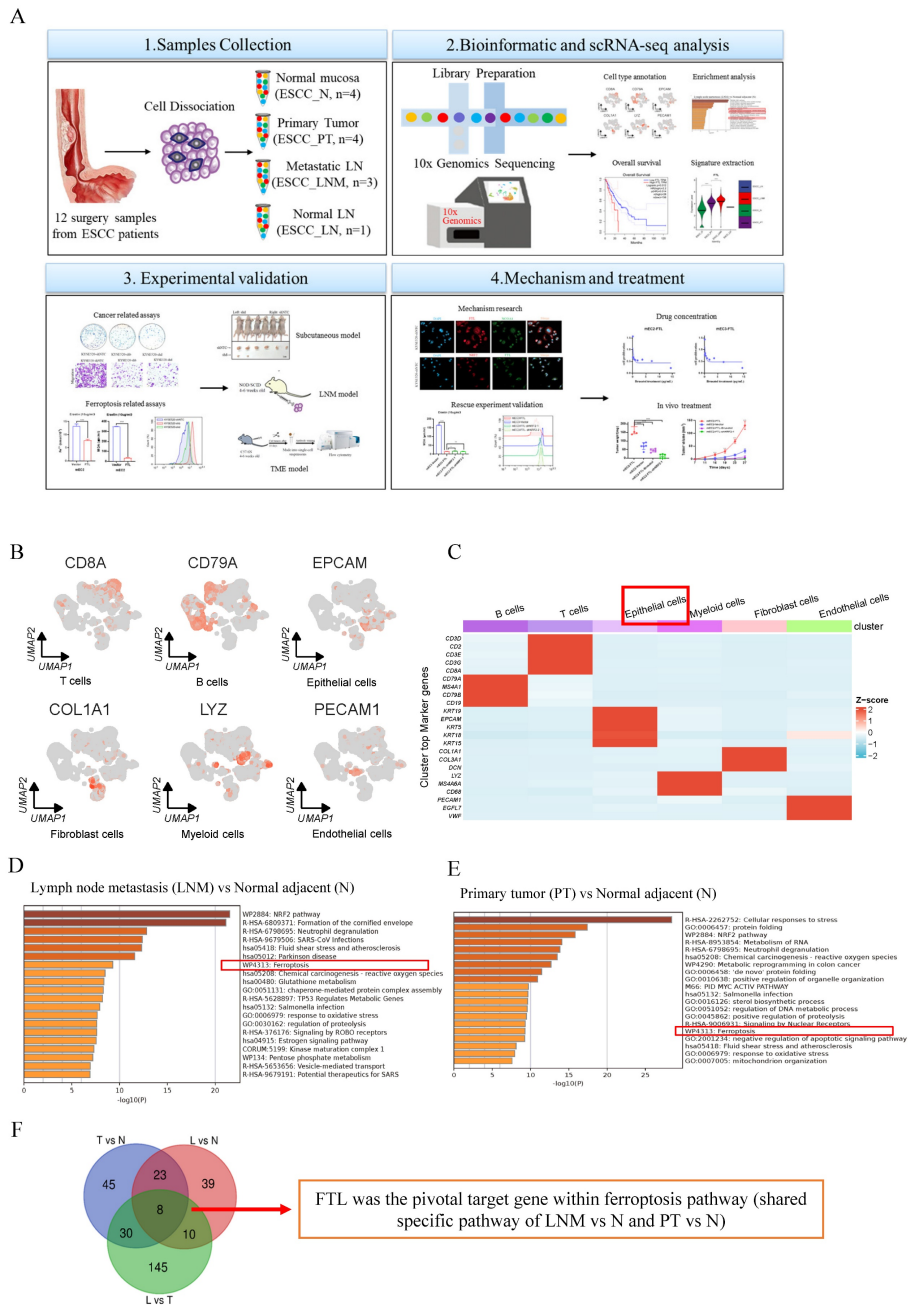
** Single-cell transcription atlas analyses of primary and metastatic ecosystems in ESCC, how FTL was selected and its bioinformatic analyses in ESCC.** (A) Diagram of our project “targeting FTL regulates ferroptosis and remodels lymph node metastasis microenvironment in esophageal squamous cell carcinoma”. (B) Umaps of representative specific marker genes, including B cells (CD79A), T cells (CD8A), epithelial cells (EPCAM), fibroblast cells (COL1A1), myeloid cells (LYZ) and endothelial cells (PECAM1). (C) A heatmap showing cell lineage-specific marker genes (rows) that are differentially expressed across ESCC cells (columns). Red: high expression and blue: low expression. (D) Enrichment heatmap of lymph node metastasis (LNM) vs normal adjacent (N) based on the single-cell transcriptional landscape of ESCC. (E) Enrichment heatmap of primary tumor (PT) vs normal adjacent (N) based on the single-cell transcriptional landscape of ESCC. (F) Venn diagram revealing how FTL was selected in this single-cell transcriptional landscape of ESCC. Statistical significances: *, P < 0.05; **, P <0.01; ***, P < 0.001.

Twelve independent surgical resected specimens were collected from four ESCC patients were fresh-processed for scRNA-seq as described previous (Figure 1A)^13^. Informed consent was obtained from all patients before the collection of samples and samples used in this study were approved by the Committees for Ethical Review of Research at Zhengzhou University (2022-KY-0149).

### Cell lines and reagents

Six ESCC cell lines (KYSE30, KYSE140, KYSE180, KYSE410, KYSE510 and KYSE520) were acquired from Deutsche Sammlung von Mikroorganismen und Zellkulturen (DMSZ, Germany)^23^. Four Chinese ESCC cell lines (EC18, and HKESC1) were obtained from Professor Srivastava and Professor Tsao from the University of Hong Kong. The mEC2 and mEC3 cell lines are two mouse ESCC cell lines derived from a single mouse ESCC tumor, different from other human ESCC cell lines (Figure S2B and S2D). Thus, we have not tested Western Blot (WB) protein expression together with other human ESCC cell lines. In addition, human embryonic kidney cell line 293T (HEK-293T cells) were purchased from American Type Culture Collection (ATCC, Manassas, Virginia, USA).

### Plasmids construction

pLenti6 expression vector made from Invitrogen was used to clone the full-length FTL isoform (NM_000146.4). Lentiviral shRNA clones for FTL were purchased from GeneCopoeia (HSH006443-LVRU6P for FTL and CSHCTR001-LVRU6P for a corresponding control) targeted FTL isoform. Lentiviral shRNA clones for NRF2 were constructed by lentiviral transduction of NRF2 shRNA via TCR2-pLKO.5-puro (Supplementary Table 1). The full-length NCOA4 isoform (NM_005437) were cloned from Genechem. We used HEK-293T packaging cells to produce related lentivirus via Lipofectamine 2000 Reagent bought from Invitrogen.

### Statistical analysis and reproducibility

Statistical analysis was performed using GraphPad Prism 8 and IBM SPSS Statistics 25, and charts were drawn. Bar chart data are expressed as mean ±SD. The box and whisker charts are expressed as median ±IQR. The violin plot is expressed as a kernel density estimate (KDE) with a median value of ±IQR. For parameter data, the p-value was tested by bilateral Student's t test. For non-parametric data from the scRNA-seq data, P-values were evaluated using a bilateral Wilcoxon signed rank test or Kruskal-Wallis univariate analysis of variance. Bilateral Pearson and Spearman correlation analysis was used to calculate the correlation and statistical significance between the two variables. In Gene Set Enrichment Analysis (GSEA), the error finding rate (FDR) and P-value are used to determine statistical significance. In survival analysis, the P-values were tested by a bilateral log-rank test. A P value less than 0.05 was considered significant. Details of the methodology can be found in the supplementary materials.

## Results

### Single-cell transcription atlas of primary and metastatic ecosystems identifies FTL as a potent oncogenic and metastatic regulator in ESCC

To comprehensively investigate the potential molecular mechanisms that develop and shape the lymph-node metastatic microenvironment of ESCC at single-cell resolution, we collected 12 independent surgical resected specimens from four ESCC patients, including primary tumor (PT) tissues, adjacent non-tumor tissues (N) and lymph node with metastasis (LNM, three cases) or without metastasis (LN, one case) for scRNA-seq^13^ (Fig. 1A). Each cluster is labeled with a specific cell type and expresses known marker genes, forming six major cell types, including B cells (CD79A, MS41, CD79B, and CD19), T cells (CD3D, CD2, CD3E, CD3G and CD8A), epithelial cells (KRT5, KRT15, KRT18, KRT19, and EPCAM), fibroblast cells (DCN, COL1A1, and COL3A1), myeloid cells (LYZ, MS4A6A, and CD68) and endothelial cells (PECAM1, EGFL7, and VWF) (Fig. 1B and S1A). The volcano plot showed high and low expression genes in different cell types, the upper genes belonged to the high expression group and the lower genes belonged to the low expression group (Fig.S1B). Epithelial cells were the key group of regulating ESCC lymph node metastasis. Therefore, epithelial cells were extracted to investigate the key genes oof ESCC development and lymph node metastasis (Fig. 1C). Through the enrichment heatmap between LNM vs N and PT vs N, we identified that ESCC and its lymph node metastasis have higher expression for pathways of ferroptosis, response to oxidative stress and glutathione metabolism (Fig. 1D and 1E). And ferroptosis was a shared specific pathway between ESCC and adjacent non-tumor tissue, as well as between ESCC lymph node metastasis and adjacent non-tumor tissue. Intersections of genes from epithelial cells between LNM vs N, PT vs N and LNM vs PT were taken and FTL was selected as the only pivotal target gene within this common pathway from the eight candidate genes shown in Figure 1G. It has its potential function related to ferroptosis in cancer just as the previous literature showed^24^ (Fig. 1F).

**Figure 2:**
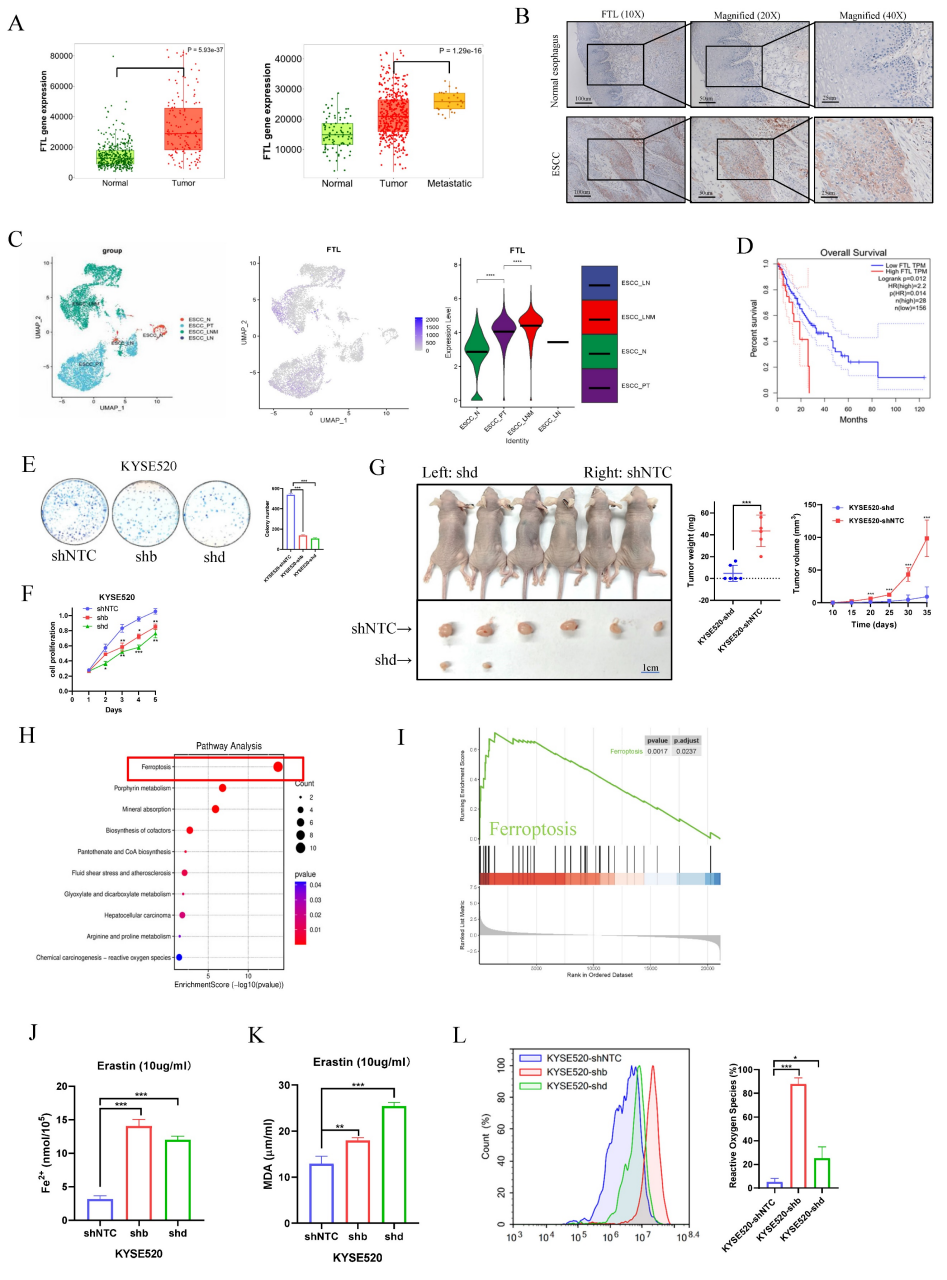
**Knockdown of FTL inhibits tumor growth and promotes ferroptosis in ESCC.** (A) Comparison of the FTL expression among non-tumor tissues, tumor and metastasis in combined TCGA and GTEx database. (B) Representative IHC staining of protein FTL in normal adjacent, primary tumor ESCC specimens. (C) The single-cell transcriptional analysis of FTL expression in normal adjacent, primary tumor, metastatic, and normal lymph node ecosystem. Figure 1C included three figures, the left one shows the distribution of single cells in different groups. The middle one illustrates how the FTL expression level was quantified in different groups via UMAP plot and the right one calculates specific statistic for FTL expression in different groups via the violin plot. (D) Kaplan-Meier survival curves showing different overall survival between with FTL high and low expression ESCC patients. (E) Foci formation assay showing different colony numbers in KYSE520 ESCC cells transfected with shNTC and FTL shRNAs. (F) XTT assay revealing cell proliferation of KYSE520 ESCC cells transfected with shNTC and FTL shRNAs. (G) *In vivo* subcutaneous implantation Nude mice model induced by 5 × 10^6^ KYSE520 ESCC cells transfected with shNTC and FTL shRNAs. KYSE520 ESCC cells transfected with shNTC and FTL shRNAs were injected subcutaneously in right and left sides of the Nude mice back respectively. Mice were sacrificed after 6 weeks for the subcutaneous tumors. Tumors of shNTC group were shown in the upper side and there were no tumors in the FTL shRNA group in the lower side. (H) GO enrichment pathways for FTL related genes. (I) GSEA analysis of ESCC FTL^high^ and FTL^low^ groups in TCGA database. (J) Iron detection assay showing the accumulation of Fe^2+^ in KYSE520 ESCC cells transfected with shNTC and FTL shRNAs. (K) MDA assay revealing the lipid formation in KYSE520 ESCC cells transfected with shNTC and FTL shRNAs. (L) ROS assay displaying the accumulation of reactive oxygen species in KYSE520 ESCC cells transfected with shNTC and FTL shRNAs. Statistical significances: *, P < 0.05; **, P <0.01; ***, P < 0.001.

Clinical data from The Cancer Genome Atlas Program (TCGA) and Genotype-Tissue Expression (GTEx) database showed the similar results and also illustrated that FTL had higher expression in metastasis than primary tumor (Fig. 2A). ESCC tissue microarray (TMA) containing tumor vs non-tumor tissues of ESCC specimens was applied to validate the expression and clinical association of FTL in ESCC. Immunohistochemistry (IHC) results displayed that FTL-expressing cells were detected more in most ESCC tissues (about 65% staining) but detected rarely (less than 5% staining) in non-tumor tissues (Fig. 2B). To further evaluate the expression of FTL in human various cancers, we used the TIMER database for analysis. FTL has higher expression in many cancers than in adjacent normal tissues especially in Esophageal carcinoma (ESCA) (Fig.S2A). Furthermore, our single-cell transcription atlas of primary and metastatic ecosystems in ESCC had also showed that FTL had higher expression in metastasis than primary tumor (Fig. 2C). Kaplan-Meier survival curves showed ESCC patients with FTL high expression had worse survival outcomes compared with patients with low expression of FTL (Fig. 2D). All of the above demonstrated that FTL was a potent oncogenic and metastatic regulator in ESCC and might present a future insight on the possible target for ESCC therapy.

### Silencing of FTL impairs cancer development and promotes ferroptosis in ESCC

To confirm whether FTL was one of the key genes for cancer development and ferroptosis in ESCC, we silenced FTL expression using shRNAs against FTL in ESCC cell lines (shb and shd). FTL expression in various ESCC cell lines was first predicted by Cancer Cell Line Encyclopedia (Fig. S2B), electrophoresis (Fig. S2C), and then detected by qPCR (Fig. S2D), western blot analysis (Fig. S2E). FTL was highly expressed in KYSE520 cell line and thus we chose this for shRNAs stable transfection. The silencing effect was detected by qPCR and western blot analysis (Fig. S2F). Next, functional assays showed knockdown of FTL significantly suppressed cells foci formation (Fig. 2E and S3A) and cell proliferation (Fig. 2F). Furthermore, the tumor growth mice model also showed that FTL knockdown decreased the tumor growth *in vivo* (Fig. 2G). All of these results verified that silencing of FTL impaired ESCC development.

FTL was reported to encode the light subunit of the ferritin protein and was the major intracellular iron storage protein in prokaryotes and eukaryotes^25^. We first used the ESCC cohort in TCGA database for detailed RNA-seq analysis. Eighty-one patients were classified into two groups according to the expression of FTL, top 25 high or low FTL expression samples were used for differential gene analyses. And the high expressed genes related to FTL was defined as the FTL related genes. Enrichment analyses in public database displayed that FTL related genes is most associated with anti-ferroptosis (Fig. 2H). GSEA analysis and ComplexHeatmap showed that clinical samples with higher FTL expression were enriched in ferroptosis (Fig. 2I), which was similar to the results of LNM vs N and PT vs N enrichment heatmap in our single-cell transcription atlas of primary and metastatic ecosystems in ESCC. This again demonstrated the importance of FTL in this model. Erastin was an activator of ferroptosis and an anti-tumor agent of cell permeability. Iron (Fe^2+^) ferrous ions assay showed that when FTL was knocked down, cancer cells would be more sensitive to erastin-induced ferroptosis (Fig. 2J). Lipid peroxidation Malondialdehyde (MDA) assay also displayed that when FTL was knocked down, the ferroptosis of cancer cells would release more lipid oxidation (Fig. 2K). Reactive oxygen species assay was studied by flow cytometry. The results showed that FTL-silenced ESCC cells released more reactive oxygen species than control group (Fig. 2L). All of the above studies proved that silencing of FTL promoted ferroptosis in ESCC.

### Overexpression of FTL promotes cancer development and inhibits ferroptosis in ESCC

**Figure 3:**
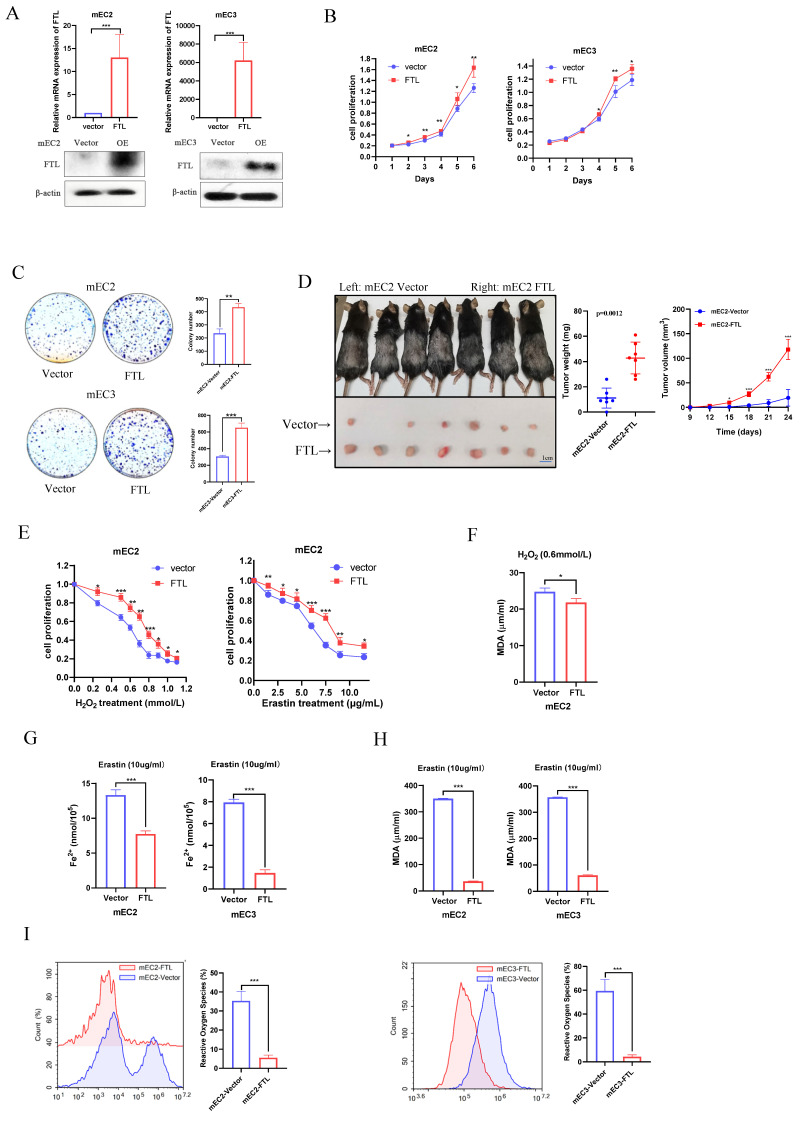
**FTL overexpression promotes tumor growth and inhibits ferroptosis in ESCC.** (A) qPCR and Western blotting of FTL in mEC2 and mEC3 cells transfected with vector and FTL isoform. β-Actin was used as a loading control. (B) XTT assay revealing cell proliferation of mEC2 and mEC3 cells transfected with lentiviruses expressing empty vector (Vector) and FTL isoform (FTL). (C) Foci formation assay showing different colony numbers in mEC2 and mEC3 cells transfected with vector and FTL isoform. (D) *In vivo* subcutaneous implantation C57/6N mice model induced by 5 × 10^6^ mEC2 transfected with vector and FTL isoform. mEC2 cells transfected with vector and FTL isoform were injected subcutaneously in left and right sides of the C57/6N mice back respectively. Mice were sacrificed after 10 days for the subcutaneous tumors. Tumors of vector group were shown in the upper side and tumors of FTL isoform group were shown in the lower side. (E) XTT assay revealing H_2_O_2_ and Erastin resistance ability of mEC2 cells transfected with vector and FTL isoform in different concentration. (F) MDA assay revealing the lipid formation in mEC2 cells transfected with vector and FTL isoform treated by H_2_O_2_. (G) Iron detection assay showing the accumulation of Fe^2+^ in mEC2 and mEC3 cells transfected with vector and FTL isoform. (H) MDA assay revealing the lipid formation in mEC2 and mEC3 cells transfected with vector and FTL isoform. (I) ROS assay displaying the accumulation of reactive oxygen species in mEC2 and mEC3 cells transfected with vector and FTL isoform. Statistical significances: *, P < 0.05; **, P <0.01; ***, P < 0.001.

FTL was reported to locate on chromosome 9 and contain only one transcript (FTL isoform). To investigate the potential role of FTL for cancer development and ferroptosis in ESCC from the opposite perspective, FTL isoform was cloned into a lentiviral vector and stably transfected into ESCC cell lines (mEC2 and mEC3). The mEC2 and mEC3 cell lines are two mouse ESCC cell lines derived from a single mouse ESCC tumor. FTL expression was validated in both mRNA and protein levels via qPCR and western blot (Fig. 3A). Next, functional results showed FTL overexpression dramatically promoted the abilities of cell proliferation (Fig. 3B), foci formation (Fig. 3C). Furthermore, the tumor growth mice model also showed that FTL overexpression significantly increased the tumor growth *in vivo* (Fig. 3D). All of these results verified that FTL overexpression enhanced ESCC development.

As analyzed in the previous section, the function of the FTL gene has been found to be closely related to ferroptosis. Oxidation resistance was investigated by Cell Proliferation Kit II (XTT) via H_2_O_2_ and erastin treatment. Results displayed that H_2_O_2_ or erastin accelerate cell death and FTL overexpressed ESCC cells resisted those induction (Fig. 3E). Also, when FTL was overexpressed, H_2_O_2_-treated cancer cells would release less lipid oxidation, while the difference between the two groups was less than that of erastin-induced ferroptosis in mEC2 cells (Fig. 3F). Iron (Fe^2+^) ferrous ions assay showed that when FTL was overexpressed, cancer cells would be more resistant to erastin-induced ferroptosis (Fig. 3G). Lipid peroxidation MDA assay also displayed that when FTL was overexpressed, erastin-induced ferroptosis of cancer cells would release less lipid oxidation (Fig. 3H). Reactive oxygen species assay was studied by flow cytometry. The results showed that when FTL overexpressed ESCC cells released less reactive oxygen species than control group (Fig. 3I). All of the above studies proved that overexpression of FTL inhibited ferroptosis in ESCC.

### FTL might affect ESCC metastasis via EMT and regulating macrophages

**Figure 4:**
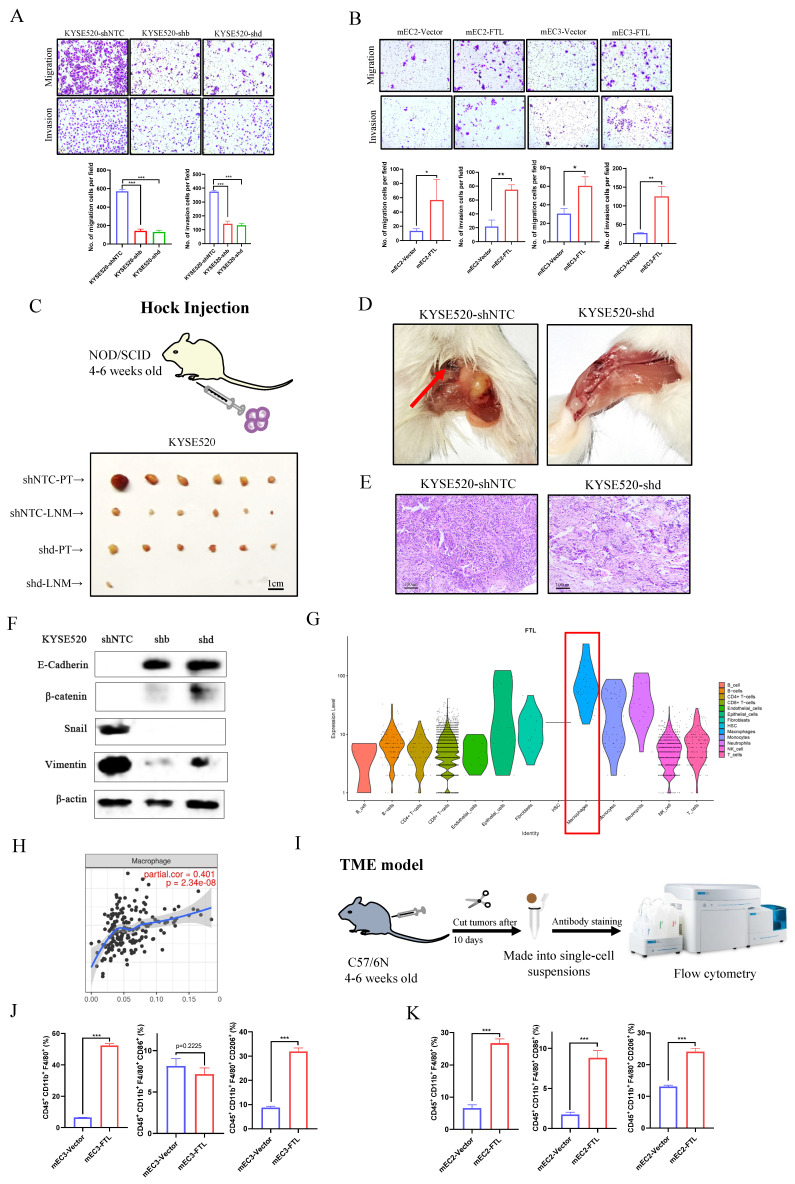
**FTL promotes ESCC migration, invasion and metastasis via EMT and macrophages.** (A) Representative images of transwell migration and matrigel invasion assays (left) and their summaries (right) in KYSE520 ESCC cells transfected with shNTC and FTL shRNAs. (B) Representative images of transwell migration and matrigel invasion assays (left) and their summaries (right) in mEC2 and mEC3 cells transfected with vector and FTL isoform. (C) *In vivo* hock injected NOD/SCID mice model induced by 1 × 10^6^ KYSE520 ESCC cells transfected with shNTC and FTL shd. Mice were sacrificed after 3 months. Panel showing mice burden with hock injected of primary tumor (PT) and lymph node metastasis (LNM). Tumors of shNTC group were shown in the upper two lines and tumors of FTL shd group were shown in the lower two lines. (D) Representative mice images of the lymph node metastasis (LNM) for hock injection mice model in shNTC and FTL shd groups. (E) Representative H&E images of the lymph node metastasis (LNM) for hock injection mice model in shNTC and FTL shd groups. (F) Western blotting of E-cadherin, β-catenin, snail and vimentin in KYSE520 ESCC cells transfected with shNTC and FTL shRNAs. β-Actin was used as a loading control. (G) FTL expression in infiltrating levels of B cells, CD8^+^T cells, CD4^+^T cells, macrophages, neutrophils and dendritic cells in ESCA. (H) Single cell analysis of FTL expression in different cell types in our single-cell transcriptional landscape of ESCC. (I) Diagram of subcutaneous implantation C57/6N mice model for ESCC microenvironment investigation. (J) *In vivo* subcutaneous implantation C57/6N mice model induced by mEC3 cells transfected with vector and FTL isoform. Subcutaneous tumors on the 10^th^ day made into single cell suspensions for flow cytometry. Flow cytometry revealing the expression of macrophages and their immunophenotypes in different groups. (K) *In vivo* subcutaneous implantation C57/6N mice model induced by mEC2 cells transfected with vector and FTL isoform. Subcutaneous tumors on the 10^th^ day made into single cell suspensions for flow cytometry. Flow cytometry revealing the expression of macrophages and their immunophenotypes in different groups. Statistical significances: *, P < 0.05; **, P < 0.01; ***, P < 0.001.

Analysis of public database data showed that FTL expression was closely related to metastasis status in ESCC patients (Fig. 2A), so we subsequently investigated the effects of FTL on cell motility and metastasis through various experiments. Cell migration and invasion assays illustrated that FTL-silenced infection significantly impaired ESCC cells motility (Fig. 4A). While, the migration and invasion abilities of ESCC cells were increased when FTL was overexpressed (Fig. 4B). For hock injection mice model (3 months after injection), only few lymph node metastases were detected in 1/6 of mice in shFTL-transfected group, whereas lymph node metastases in the groin of the mice were observed in 6/6 of mice in shNTC-transfected group (Fig. 4C and 4D). Hematoxylin and Eosin (H&E) staining further confirmed lymph node metastasis (Fig. 4E). Above all, these results validated that FTL promoted ESCC lymph node metastases. Then, WB was performed to explore epithelial-mesenchymal transition (EMT) related markers for further mechanism exploration (Fig. 4F). The results showed that silencing of FTL upregulated epithelial marker E-cadherin, β-catenin and downregulated mesenchymal markers Vimentin and Snail, illustrated that FTL promoted EMT in ESCC cells.

Single cell data in the Human Protein Atlas also illustrated that macrophages were of great significance to FTL gene function (Fig. S3B and S3C). In order to double check that point, we used our single-cell transcription atlas of primary and metastatic ecosystem to explore FTL expression in all immune cells infiltration and found similar results (Fig.4G). TIMER tool was used to explore relationship between FTL and various immune cells infiltration and it showed that FTL was most relevant to macrophages (Fig. 4H). We speculated that whether it was related to the formation of immune microenvironment in the early stage of ESCC metastasis. Thus, we used the subcutaneous injection model in C57/6N mice (immunocompetent mice) for immune microenvironment exploration, 5 × 10^6^ FTL transfected and control transfected cells were injected subcutaneously into different sides of mice back. Mice were sacrificed on the 10th day for its tumors. Then, the tumors were made into single-cell suspensions for flow cytometry analyses respectively. For mEC3 cells, it was obviously that FTL transfected cells recruited more macrophages (CD45^+^ CD11b^+^ F4/80^+^ marked cells) than the control group. Also, FTL transfected cells recruited fewer M1 type macrophages (CD45^+^ CD11b^+^ F4/80^+^ CD86^+^ marked cells) than the control group (although not statistically significant), yet more M2 type macrophages (CD45^+^ CD11b^+^ F4/80^+^ CD206^+^ marked cells) than the control group (Fig. 4I and 4J). For mEC2 cells, it had similar results except for M1 type macrophages (Fig.4K). The results implied that FTL promotes ESCC metastasis by recruit more macrophages. This discrepancy in results between the cell lines may be attributed to the heterogeneity of the cell lines used. Since the effect of FTL on M2 polarization of TAMs is controversial between mEC2 and mEC3cells, we could not conclude that FTL regulate the M2 polarization of TAMs. The lack of consistent results across multiple cell lines led to a decision not to further investigate the mechanism by which FTL induces M2 macrophages.

### FTL promotes ESCC development and metastasis via NRF2 pathway and inhibits ferroptosis via NCOA4 protein

**Figure 5:**
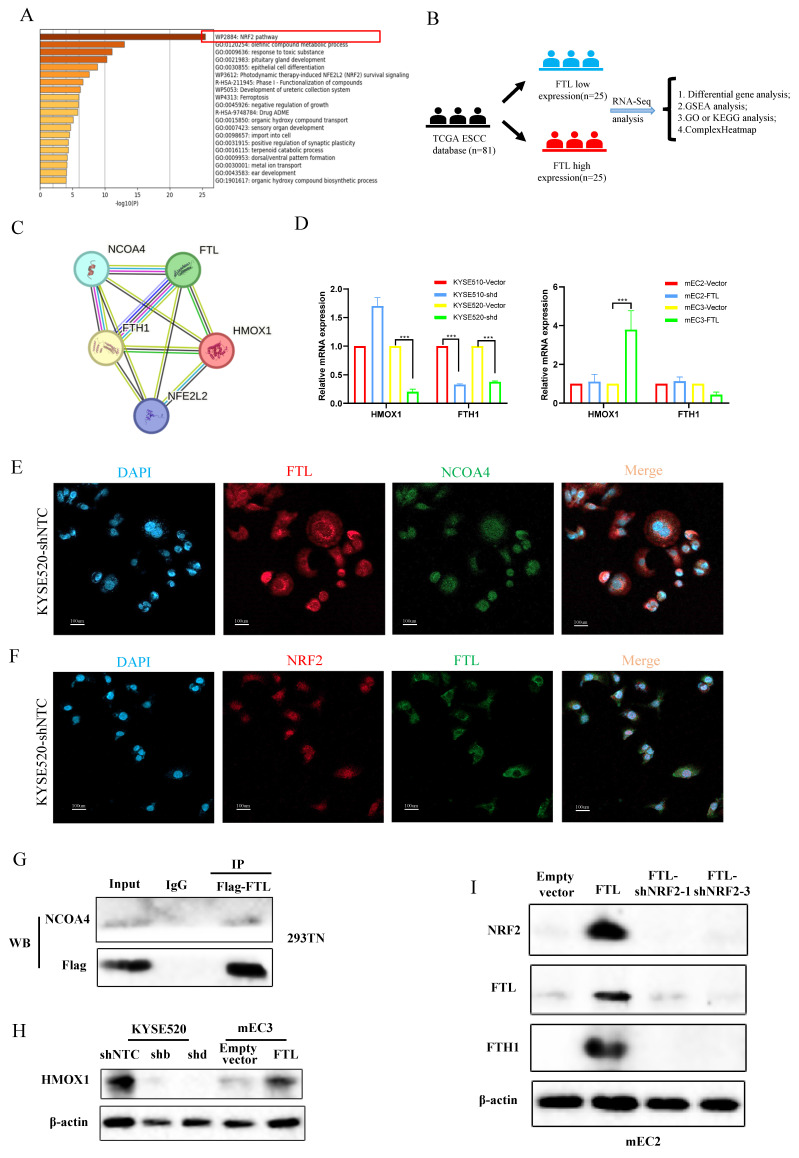
** FTL promotes ESCC development and metastasis via NRF2 pathway and inhibits ferroptosis via NCOA4 protein.** (A) Metascape analyses for ESCC FTL^high^ and FTL^low^ groups in ESCC single-cell transcriptional landscape. (B) Diagram of exploring upstream and downstream genes of FTL in TCGA database. (C) Protein-protein interaction network of FTL according to NRF2 and ferroptosis-related proteins. (D) qPCR of HMOX1, FTH1 in KYSE510 and KYSE520 ESCC cells transfected with shNTC and FTL shRNA. qPCR of HMOX1, FTH1 in mEC2 and mEC3 cells transfected with vector and FTL isoform. (E) IF double staining of FTL and NCOA4 in KYSE520 ESCC cells transfected with shNTC and nuclei stained with DAPI. (F) IF double staining of FTL and NRF2 in KYSE520 ESCC cells transfected with shNTC and nuclei stained with DAPI. (G) Cell lysates from HEK-293T cells transfected with FTL-Flag plasmids subjected to immunoprecipitation (IP) with NCOA4 antibody or control immunoglobulin G (IgG). (H) Western blotting of HMOX1 in KYSE520 ESCC cells transfected with shNTC, FTL shRNAs and mEC3 cells transfected with vector, FTL isoform. β-Actin was used as a loading control. (I) Western blotting of NRF2, FTL, FTH1 in mEC2 cells transfected with vector, FTL isoform, FTL-NRF2 shRNAs. β-Actin was used as a loading control. Statistical significances: *, P < 0.05; **, P <0.01; ***, P < 0.001.

To further explore the molecular mechanism of FTL function regulation, we used Metascape to analyze ESCC single-cell transcriptional landscape in ESCC FTL^high^ and FTL^low^ groups (Fig. 5A). The results displayed that FTL^high^ group were enriched in NRF2 and ferroptosis signaling pathways. Also, we used the ESCC cohort in TCGA database for detailed RNA-seq analysis. Eighty-one patients were classified into two groups according to the expression of FTL, top 25 high or low FTL expression samples were used for differential gene analyses (Fig. 5B). DESeq2, edgeR and limma package were used for RNA-seq analysis respectively. GSEA analysis showed highly expressed signaling pathways (Fig. S4A) and volcano plots displayed high or low expression genes in the comparison of FTL^high^ and FTL^low^ groups from different algorithms (Fig.5SA). At the same time, we have extensively reviewed the relevant literatures on the mechanism of ferroptosis, and found that NRF2 has been found to affect the expression of ferritin (FTL) in tumors^26^. Moreover, the ferroptosis function of FTL as ferritin was closely related to the interaction of NCOA4 and the regulation of HO-1 and FTH1 proteins^27^,^28^. STRING was used for those key protein interaction exploration (Fig. 5C). According to the above methods, we collected some key genes, which were highly related to oxidative stress and ferroptosis, for qPCR verification (Fig.5D). We found that the variation of FTL expression level regulated the expression of ferroptosis-related FTH1/HMOX1 axis (Fig.5C, 5H and 5I). Immunofluorescence was used to explore the co-expression of FTL with NRF2 and NCOA4 (Fig. 5E and 5F). The interaction between FTL and NCOA4 was confirmed via immunoprecipitation experiment and it showed that NCOA4 was bound with FTL in HEK-293T cells (Fig. 5G).

### Silencing of NRF2 reverses FTL promoting cancer development in ESCC

Given that NRF2 has been well characterized to promote cancer development and positively regulate oxidative stress^29^, we silenced NRF2 expression with two shRNAs against NRF2 in FTL-overexpression ESCC cell lines. Western blot was used to confirm NRF2 expression in protein levels (Fig. 5I). It also displayed that FTL expression was significantly decreased when NRF2 was knocked down in FTL-overexpressed mEC2 cells (Fig. 5I). Furthermore, FTH1 had the corresponding transformation (Fig. 5I).

**Figure 6:**
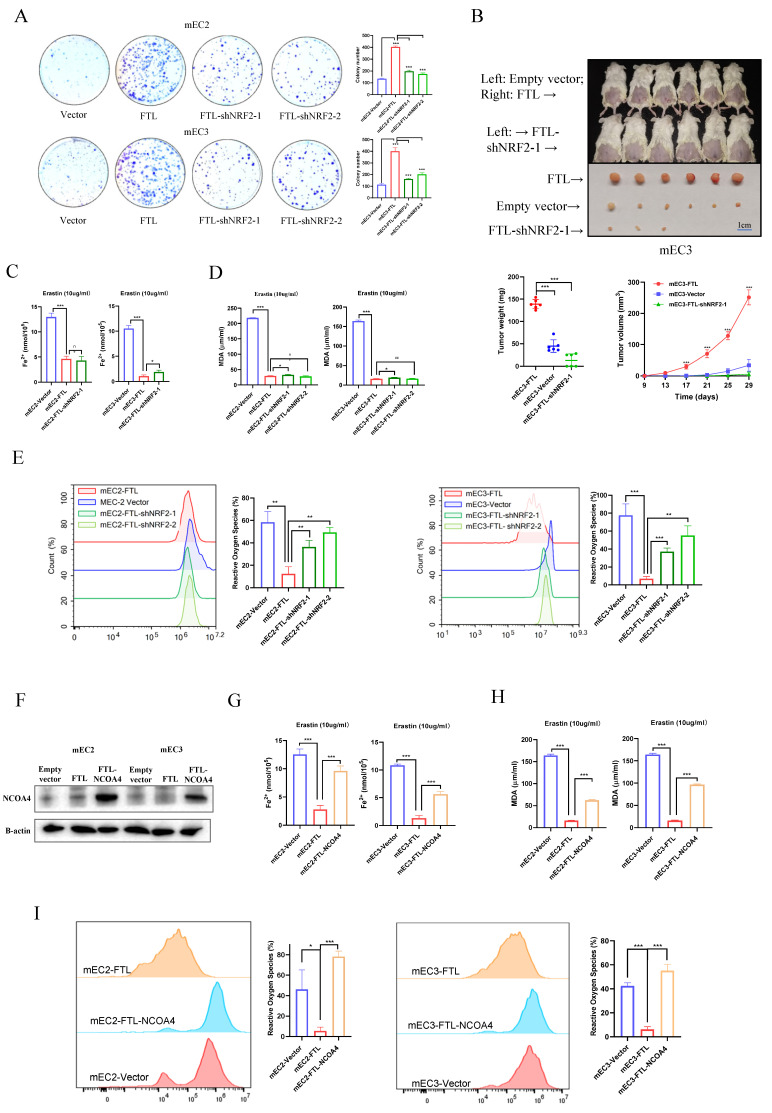
** Downregulation of NRF2 or upregulation of NCOA4 impairs FTL oncogenic and ferroptosis effects in ESCC.** (A) Foci formation assay showing different colony numbers in mEC2 and mEC3 cells transfected with vector, FTL isoform, FTL-NRF2 shRNAs. (B) *In vivo* subcutaneous implantation NOD/SCID mice model induced by 5 × 10^6^ mEC3 cells transfected with vector, FTL isoform, FTL-NRF2 shRNA. Mice were sacrificed after 4 weeks for the subcutaneous tumors. Tumors of FTL isoform group were shown in the upper side, tumors of vector group were shown in the middle side and tumors of FTL-NRF2 shRNA group were shown in the lower side. For the first line of mice, FTL-overexpressing cells and empty vector control cells were injected into right and left side, respectively. (C) Iron detection assay showing the accumulation of Fe2+ in mEC2 and mEC3 cells transfected with vector, FTL isoform, FTL-NRF2 shRNAs. (D) MDA assay revealing the lipid formation in mEC2 and mEC3 cells transfected with vector, FTL isoform, FTL-NRF2 shRNAs. (E) ROS assay displaying the accumulation of reactive oxygen species in mEC2 and mEC3 cells transfected with vector, FTL isoform and FTL-NRF2 shRNAs. (F) Western blotting of NCOA4 in mEC2 and mEC3 cells transfected with vector, FTL isoform, FTL-NCOA4 isoform. β-Actin was used as a loading control. (G) Iron detection assay showing the accumulation of Fe2+ in mEC2 and mEC3 cells transfected with vector, FTL isoform, FTL-NCOA4 isoform. (H) MDA assay revealing the lipid formation in mEC2 and mEC3 cells transfected with vector, FTL isoform, FTL-NCOA4 isoform. (I) ROS assay displaying the accumulation of reactive oxygen species in mEC2 and mEC3 cells transfected with vector, FTL isoform, FTL-NCOA4 isoform. Statistical significances: *, P < 0.05; **, P <0.01; ***, P < 0.001.

Next, functional results showed NRF2 knockdown significantly reversed the abilities of FTL enhancing cell foci formation in mEC2 and mEC3 cells (Fig. 6A). Also, the tumor growth mice model showed that NRF2 knockdown impaired FTL increasing tumor growth *in vivo* (Fig. 6B). Iron (Fe^2+^) ferrous ions assay showed that when NRF2 was knocked down, cancer cells had similar resistance to erastin-induced ferroptosis compared with FTL overexpressed group (Fig. 6C). Lipid peroxidation MDA assay also displayed that when NRF2 was knocked down, cancer cells had similar resistance to erastin-induced ferroptosis compared with FTL overexpressed group (Fig. 6D). Reactive oxygen species assay was studied by flow cytometry. Yet, it showed that when NRF2 was knocked down, ESCC cells released more reactive oxygen species than control group (Fig. 6E). Taken together, these findings suggested that NRF2 reversed overexpressed FTL function of enhancing cancer development and slightly affects ferroptosis in ESCC.

### Overexpression of NCOA4 reverses FTL inhibiting ferroptosis in ESCC

Given that NCOA4 has been well characterized to promote ferroptosis^30^, NCOA4 isoform was cloned into a lentiviral vector and stably transfected into FTL overexpressed ESCC cell lines. Western blot was used to confirm NCOA4 expression in protein levels (Fig. 6F). Next, functional assays were performed in NCOA4-overexpressed FTL-overexpressed ESCC cells, and results illustrated that overexpression of NCOA4 reversed the abilities of FTL enhancing cell foci formation in mEC2 and mEC3 cells (Fig. S6A). Iron (Fe^2+^) ferrous ions assay showed that when NCOA4 was overexpressed, cancer cells were more resistant to erastin-induced ferroptosis compared with only FTL overexpressed group (Fig. 6G). Lipid peroxidation MDA assay also displayed that when NCOA4 was overexpressed, cancer cells had more resistance to erastin-induced ferroptosis compared with only FTL overexpressed group (Fig. 6H). Reactive oxygen species assay was studied by flow cytometry. Results showed that when NCOA4 was overexpressed, ESCC cells released more reactive oxygen species than only FTL overexpressed group (Fig. 6I). Taken together, all the above illustrated that NCOA4 reversed overexpressed FTL function of enhancing cancer development and inhibiting ferroptosis in ESCC.

### Silencing of NRF2 reverses FTL promoting ESCC metastasis via EMT and macrophages

**Figure 7:**
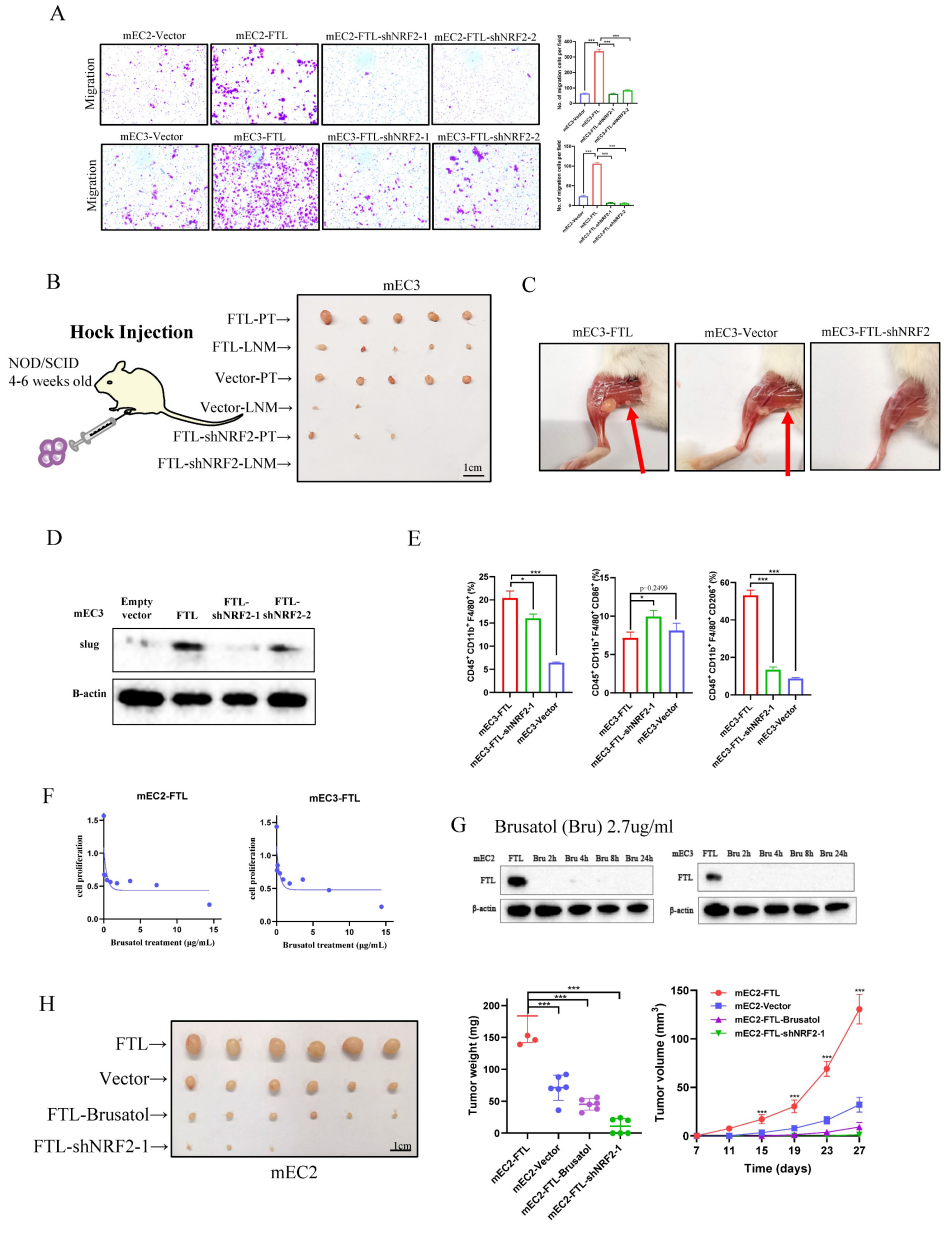
** Downregulation of NRF2 impairs FTL metastasis effects in ESCC.** (A) Representative images of transwell migration assays (left) and their summaries (right) in mEC2 and mEC3 cells transfected with vector, FTL isoform and FTL-NRF2 shRNAs. (B) *In vivo* hock injected NOD/SCID mice model induced by 1 × 10^6^ mEC3 cells transfected with vector, FTL isoform and FTL-NRF2 shRNA. Mice were sacrificed around 70 days. Panel showing mice burden with hock injected of primary tumor (PT) and lymph node metastasis (LNM). Tumors of FTL isoform group were shown in the first two lines, tumors of vector group were shown in the middle two lines and tumors of FTL-NRF2 shRNA group were shown in the last two lines. (C) Representative mice images of the lymph node metastasis (LNM) for hock injection mice model in vector, FTL isoform groups. (D) Western blotting of slug in mEC2 cells transfected with vector, FTL isoform, FTL-NRF2 shRNAs. β-Actin was used as a loading control. (E) *In vivo* subcutaneous implantation C57/6N mice model induced by mEC3 cells transfected with FTL isoform and FTL-NRF2 shRNAs. Subcutaneous tumors on the 10th day made into single cell suspensions for flow cytometry. Flow cytometry revealing the expression of macrophages and their immunophenotypes in different groups. (F) IC50 of Brusatol in FTL-overexpression different ESCC cell lines. (G) Western blotting of FTL-overexpression ESCC cell lines treated Brusatol after 0, 2, 4, 8, 24h for the recommended dose concentration. β-Actin was used as a loading control. (H) *In vivo* subcutaneous implantation Nude mice model induced by 5 × 10^6^ mEC2 cells transfected with vector, FTL isoform, FTL isoform treated with Brusatol and FTL-NRF2 shRNAs. Mice were sacrificed after 4 weeks for the subcutaneous tumors. Tumors of FTL isoform group were shown in the first line, tumors of vector group were shown in the second line, tumors of FTL isoform treated with Brusatol group were shown in the third line and tumors of FTL-NRF2 shRNA group were shown in the last line. Statistical significances: *, P < 0.05; **, P <0.01; ***, P < 0.001.

As the results above showed that NRF2 reversed overexpressed FTL function of enhancing cancer development, we further explored whether it could reverse FTL promoting metastasis. Cell migration assays showed that when NRF2 was knocked down in FTL-overexpressed ESCC cells, it significantly impaired ESCC cells motility (Fig. 7A). For *in vivo* hock injection mice model, 3 months after hock injection of mEC3 cells, no lymph node metastases were observed in 0/5 of mice in shNRF2-FTL-overexpressed group, whereas lymph node metastases in the groin of the mice were detected in 5/5 of tested mice in FTL-overexpressed group (Fig. 7B and 7C). H&E staining further confirmed lymph node metastasis (Fig. S7A). In all, these results implied that NRF2 reversed FTL promoting ESCC metastasis. Also, western blot was performed to explore epithelial-mesenchymal transition (EMT) related markers (Fig. 7D). The results showed that silencing of NRF2 reversed upregulated epithelial marker Slug, illustrated that NRF2 reversed FTL promoting ESCC metastasis via EMT.

According to our previous studies, we found that FTL regulated macrophages to promote the early immune microenvironment of ESCC metastasis. We further explored whether NRF2 could reverse metastasis via macrophages. Thus, we used the subcutaneous injection model in C57/6N mice (immunocompetent mice) for immune microenvironment exploration, 5 × 10^6^ FTL transfected and control transfected cells were injected into different sides of mice back subcutaneously. Mice were sacrificed on the 10th day for its tumors. Then, the tumors were made into single-cell suspensions for flow cytometry analyses respectively. For mEC3 cells, it was obviously that shNRF2-FTL-overexpressed mEC3 cells recruited fewer macrophages (CD45^+^ CD11b^+^ F4/80^+^ marked cells) than FTL-overexpressed group. Also, FTL transfected cells recruited fewer M2 type macrophages (CD45^+^ CD11b^+^ F4/80^+^ CD206^+^ marked cells) than FTL-overexpressed group (Fig. 7E). The results implied that NRF2 reversed FTL promoting ESCC metastasis via the downregulation of macrophages recruitment.

### Brusatol reverses FTL promoting cancer development in ESCC

**Figure 8:**
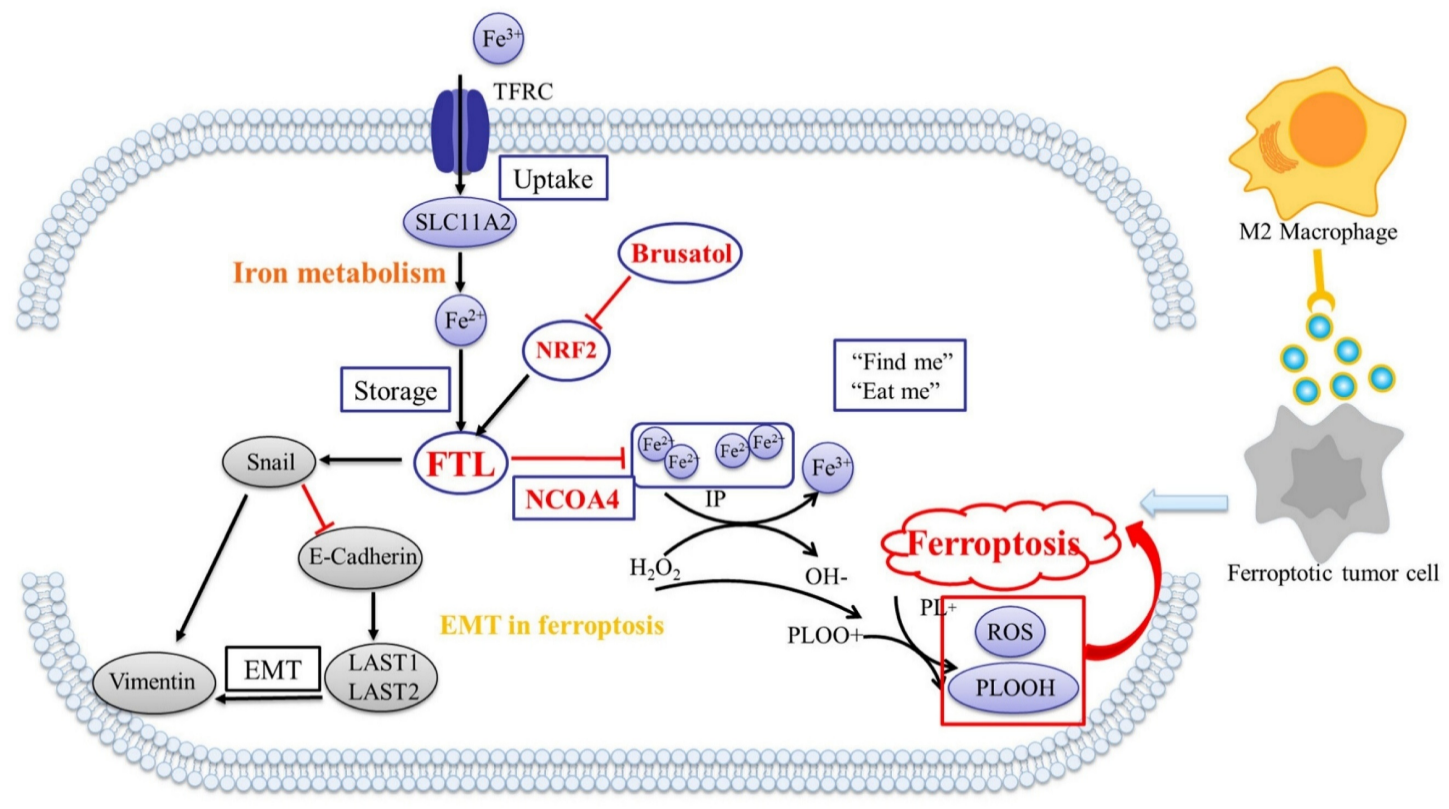
** Summary of regulatory mechanisms for FTL in ESCC.** (A) Mechanism of Brusatol silenced NRF2 and reversed FTL promoting ESCC development and metastasis via EMT and macrophages.

The treatment of tumors via traditional Chinese medicine (TCM) is an important way to develop new drugs, and there are many studies showing the effects of Brucea javanica on tumor development, metastasis and ferroptosis^31^,^32^. Brusatol is a diterpenoid, a critical component extracted from the Brucea javanica plant^33^, which has been shown to inhibit NRF2 activity in previous literatures^34^, ^35^. It displayed that Brusatol significantly reduced the expression of FTL protein, and the overexpression of this gene was reversed in just two hours. IC50 of Brusatol in FTL-overexpression ESCC cell lines was checked via XTT assay (Fig. 7F). To check whether Brusatol affect FTL expression, western blot was used to check FTL-overexpression ESCC cell lines treated Brusatol after 0, 2, 4, 8, 24h for the recommended dose concentration (Fig. 7G). Also, the tumor growth mice model showed that knockdown of NRF2 and Brusatol significantly reversed FTL increasing tumor growth *in vivo* (Fig. 7H). Collectively, our findings suggested that Brusatol silenced NRF2 and then reversed FTL promoting ESCC development and metastasis via EMT and macrophages (Fig. 8A).

## Discussion

Although radical surgery could cure esophageal cancer, more than half of ESCC patients are initially diagnosed at an advanced stage, and such patients cannot undergo surgery due to extensive metastasis. Local invasion and distant metastasis are the main reasons for the low 5-year survival rate in ESCC patients^2^,^5^. Studies have shown that the plasticity of tumor cells enables metabolic reprogramming that recruits and modifies cells to make them more likely to metastasize, and that this metabolic reprogramming is associated with tumor cells' sensitivity to ferroptosis^9^, ^36^. Thus, the suitable ferroptosis targets as to this perspective is expected to develop drugs to treat ESCC metastasis. Our lab has reported the single-cell transcriptome of patients with ESCC lymph node metastasis and generated a tumor microenvironment (TME) map of the entire ESCC metastasis^13^, and we found that targeting FTL might regulate the remodeling of ferroptosis and lymph node microenvironment in ESCC.

Ferritin light chain (FTL) is a gene encoding the light subunit of ferritin. Ferritin is an iron storage protein composed of 24 subunits of heavy and light ferritin chains^14^,^15^. In the past, FTL has been linked to diseases caused by abnormal iron levels, such as Hyperferritinemia-cataract syndrome and Neuroferritinopathy^37^, ^38^. It was not until 2016 that a study found that FTL expression was increased in glioblastoma and acute myeloid leukemia, and silencing FTL could inhibit the proliferation of tumor cells^17^,^18^. Later, a small number of studies have found that FTL affects the chemotherapy resistance of tumor cells, the occurrence of EMT and the promotion of metastasis in solid tumors such as colorectal cancer and liver cancer^19^, ^39^. However, there is no research of FTL in the progress of ESCC, and our study just fills the gap in this part.

Epithelial cells were extracted from scRNA data of patients with ESCC lymph node metastasis. Key genes related to ferroptosis in ESCC progression and lymph node metastasis were identified, and only FTL gene met the requirements. Both public databases and our single cell atlas showed that FTL expression was higher in tumors than in neighboring normal tissues, and patients with high FTL expression had shorter overall survival (OS). The IHC also confirmed this conclusion. To explore the role of FTL in ESCC development and progression, the functions of FTL-overexpressed cells and FTL-knockdown cells were measured *in vitro* and *in vivo*. The results showed that FTL has a strong carcinogenic ability, could promote cell growth *in vitro*, promote tumor formation *in vivo*, and significantly reduce the sensitivity of ESCC cells to ferroptosis. In addition, the silencing of FTL affected the expression of EMT-related genes in ESCC cells, such as E-Cadherin and snail^40^, ^41^, and inhibited ESCC cells invasion and migration *in vitro* and lymph node metastasis of ESCC cells *in vivo*. At the same time, the public database and single cell atlas also showed that FTL function were closely related to macrophages. By simulating the tumor immune microenvironment in C57 mice, we found that FTL promoted metastasis by recruiting more macrophages^42^.

Next, we studied the mechanism of FTL including its related genes and pathways, and explored the significance of their network on the TME remodeled by ferroptosis. NRF2 (nuclear factor erythroid 2-related factor 2) is a transcription factor that regulates the expression of various cell-protective genes^43^. NRF2-dependent ESCC demonstrates enhanced malignancy and the development of resistance to chemoradiotherapy^44^. NRF2 has been reported to play roles in regulating redox homeostasis, lipid metabolism, and ferroptosis in tumors. It could bind to antioxidant response elements (AREs) in the promoter region of ferritin (such as FTL) and lipid metabolism-related genes. Once activated, NRF2 will translocate into the nucleus, where it interacts with these AREs, promoting the transcription of downstream ferritin and lipid metabolism-related protein levels^45^. Our experimental results showed that NRF2 knockdown reversed the carcinogenic ability of FTL, inhibited the growth of FTL-overexpressed cells *in vitro*, and the formation of FTL-overexpressed tumors *in vivo*. In addition, NRF2 reversed FTL overexpression affecting certain EMT-related proteins expression in ESCC cells. Simultaneously, NRF2 inhibited the promotion of FTL in ESCC metastasis *in vivo* and *in vitro*, and recruited more macrophages in TME. Besides, overexpression of NCOA4 significantly increased the sensitivity of ESCC cells to ferroptosis. Finally, we found that Brusatol, an effective ingredient of Brucea javanica, effectively targeted FTL and NRF2 and inhibited the formation of FTL overexpression tumors *in vivo* and *in vitro*.

### Limitations of the study

In our study, we used totally three cell lines to verify its functional assays *in vitro*. While these cell lines were carefully selected based on their relevance to ESCC research, the narrow range of cellular models inherently imposes constraints on the generalizability and robustness of the findings. Another significant limitation of this study lies in the fact that the scale of the cohort we verified in the *in vivo* mice model experiments was relatively small, with only 5-6 mice in each experimental group. The limited sample size introduced significant constraints, affecting the reliability, universality and statistical robustness of our results. Another key limitation of our research lies in the insufficient depth of the mechanism when analyzing the regulatory relationship among NRF2, NCOA4 and FTL. This is mainly because these interactions have been widely described in the previous literatures. Although our research aims to investigate their roles in specific biological contexts (for example, disease phenotypes or treatment responses), we have not fully revealed the complex molecular mechanisms that underpins their crosstalk, which limits new insights into their functional interdependencies.

## Conclusion

In conclusion, FTL was defined as a key target that regulated the metastasis microenvironment of ESCC lymph nodes via ferroptosis, and it could promote tumorgenicity and metastasis of ESCC cells by regulating macrophages and affecting EMT protein changes. Silencing NRF2 down-regulated the expression of FTL in ESCC, adjusted the recruitment of macrophages and influenced the changes of EMT protein to inhibit tumorgenicity and metastasis. While, NCOA4 exerted its function of inhibiting tumor development by affecting FTL to regulate the ESCC sensitivity to ferroptosis. Finally, we found that Brusatol, an active ingredient in Brucea javanica, targeted FTL and NRF2, which might be a brand-new therapeutic strategy for patients with advanced ESCC.

## Supplementary Material

Supplementary methods, figures and tables.
