# Supplementary material for: Targeting FTL regulates ferroptosis and remodels lymph node metastasis microenvironment in esophageal squamous cell carcinoma
Source: Int J Biol Sci. 2025 Oct 16;21(15):6616–32. doi: 10.7150/ijbs.112017 (PMC12631076; doi:10.7150/ijbs.112017)
Supplement: Supplementary file 1 — Supplementary methods, figures and tables. [file ijbsv21p6616s1.pdf]

SUPPLEMENTARY INFORMATION

Targeting FTL regulates ferroptosis and remodels lymph node metastasis microenvironment in esophageal squamous cell carcinoma

Supplementary materials and methods

Preparation for single-cell data

The gene expression matrix metadata was converted to a Seurat object using Seurat R package (v 4.0). To remove low quality cells, the various metrics distribution of the data was visualized and the following thresholds were applied: nUMI > = 300, nGene > = 200, log10GenesPerUMI > 0.8 and mitoRatio < 0.2. Doublets were removed by DoubletFinder (v 2.0). From the remaining cells, the gene expression dataset was normalized, scaled, and subsequently dimensionally reduced. Major cell lineages, including epithelial cells, T cells, B cells, myeloid cells, fibroblasts, and endothelial cells, were projected on the two dimensional UMAP representation and annotated using well recognized signatures. FindMarkers were used to compare differentially expressed genes between different groups. Venn plot showed the intersection of differentially expressed genes from different comparisons.

Preparation for public RNA-seq datasets

ESCC cohort in TCGA database were collected for detailed RNA-seq analysis. Eighty-one patients were classified into two groups according to the expression of FTL, top 25 high or low FTL expression samples were used for differential gene analyses. And the high expressed genes related to FTL was defined as the FTL related genes for the GO and GSEA analyses. In Gene Set Enrichment Analysis (GSEA), the error finding rate (FDR) and P-value are used to determine statistical significance. Genotype-Tissue Expression (GTEx) database was an online analysis database for

different genes expression in normal, tumor and metastasis group.

Cell culture

All cell lines were maintained in a humidified incubator at 37°C containing 5% CO^2. In everyday cultivation, RPMI-1640 Medium (Gibco BRL, Grand Island, NY, USA) supplemented with 1% 200mM L-glutamine (Gibco BRL. Grand Island. NY. USA) and 10% fetal bovine serum (FBS) (Gibco BRL, Grand Island, NY, USA) was used for the six ESCC cell lines (KYSE30, KYSE140, KYSE180, KYSE410, KYSE510 and KYSE520). Sometimes we would withdraw or reduce FBS. High glucose Dulbecco's Modified Eagle Medium (Gibco BRL, Grand Island, NY, USA) supplemented with 1% 200mM L-glutamine (Gibco BRL, Grand Island, NY, USA) and 10% FBS (Gibco BRL, Grand Island, NY, USA) was used to support the other cell lines (EC18, HKESC1, HEK-293T).

Lentivirus production

For lentivirus production, HEK-293T packaging cells were transfected with lentiviral vectors using Lipofectamine 2000 Reagent (Invitrogen). After 72 h of incubation, the viral supernatant was collected and filtered. ESCC cells were incubated overnight with the viral supernatant and supplemented with 10 μg/ml polybrene. Stable clones were selected using puromycin and blasticidin.

Western blotting and qPCR

Denatured protein samples with molecular weight markers were packed into 8-12% SDS-PAGE gel Wells. After electrophoresis, the protein was transferred to PVDF membrane at 200A constant current for 2h. The PVDF membrane was sealed in TNS-T with 5% skim milk for more than 1 h and incubated overnight with primary antibody at 4℃. After TBS-T washing, PVDF membrane was incubated with the corresponding secondary antibody for 1 hour. Immunoassays were performed using ECL Western Blotting Detection Kit and Hyperfilm (Amersham Biosciences, Piscataway, NJ). Total RNA was extracted via TRIZOL Reagent (Invitrogen), and the

cDNA was synthesized from 4ul of total RNA by PrimeScript RT Master Mix (Takara Bio). Quantitative real-time PCR (qPCR) was performed using the TB Green Premix Ex Taq II (TAKARA) and a LightCycler® 480 System (ROCHE). The mRNA expression of FTL, GPX2, GPX4, TXNRD1, NQO1, PRDX1, SLC40A1, GSTM3, GSTM4, HMOX1, FTH1 and β-Actin was assessed by qPCR with the primers (Supplementary Table 2).

XTT cell proliferation assay

The cells were planted in 96-well plates at the confluence point of 1×10^3 cells per well. Cell proliferation XTT kit (Roche Applied Science, Penzberg, Germany) assays cell proliferation rate according to manufacturer's instructions. Each cell line was triploid sown and measured daily. Curves are represented by three averages. The two sets of data at each time point were statistically analyzed by t-test, and the results were obtained by comparing multiple sets of data.

Cell proliferation and drug resistance assay

In vitro cell proliferation rate was assessed by monitoring the cell number for 5–7 days in cell culture. The cells in each condition were seeded at 1,000 cells/well for proliferation assay or 8,000 cells/well for drug resistance assay in 96-well in triplicate and incubated in a 37 °C humidified CO_2 incubator with the drug or vehicle containing medium refreshed every other day. The number of cells was determined by XTT kit (Roche Diagnostics, Indianapolis, IN).

Migration, and invasion assay

Migration and invasion assays were determined to evaluate cell motility (BD Biosciences, San Jose, CA). Matrigel was used in invasion assays. Approximately 8×10^4cells were seeded in the medium without FBS on transwell upper chambers, and the lower chamber was supplied with a medium of complete 10% FBS. After 24-48 h, the lower membrane surface was stained using crystal violet to count the cell number under a microscope.

Immunohistochemistry (IHC)

Standard streptavidin-biotin-peroxidase complex method was used for immunohistochemistry. Soon, the tissue sections were dewaxed with xylene and then rehydrated with a reduced concentration of ethanol. Endogenous peroxidase was blocked with H_2O_2 (0.3%) for 10 min. Tissue slides were cultured in a 10 mM citric acid buffer at 98℃ for 10-20 min and cooled for several hours or antigen recovered. After the slices were taken, the tissue slides were sealed with 10% normal goat serum for more than 1 h and incubated overnight with primary antibody at 4℃. On the second day, the tissue sections were rinsed with PBS 4 times for 15 min each, and incubated with secondary antibody at 37℃ for 30 min. Incubate with hematoxylin and prepared DAB (DAKO, Carpinteria, CA) for 3-10 seconds and stain the nucleus.

Immunofluorescence (IF)

Slides containing adherent cells or retrieved tissues were fixed with 4% paraformaldehyde for 10 min. In some cases, such as nucleoprotein staining, 1-5 minutes (optional) of infiltration is performed using 0.05% Triton X-100. The slides were blocked with protein blocks (DAKO, Carpinteria, CA) for 10 min. After washing by TBS, the slides were loaded with primary antibody and incubated overnight at 4℃. On the second day, the primary antibody was rinsed with TBS, and the slides were incubated with the fluorophore-coupled secondary antibody in a dark and humid environment for 30 min. Finally, DAPI stain the nucleus (Invitrogen, Carlsbad, CA).

Iron assay

Intracellular ferrous iron (Fe^{2+}) level was determined using the iron assay kit (E-BC-k733-M) purchased from Elabscience according to the manufacturer's instructions. Cells were seeded onto 10 cm plate (5×10^6 cells per plate) and treated with corresponding drugs for 24 h. Cells were collected and washed in ice-cold PBS and

homogenized in iron assay buffer 1#, Ultrasonic crushing 4 °C, then centrifuged (10,000 × g, 10 min) at 4 °C. Collect the supernatant and add iron assay buffer 2# to each sample, mix, and incubate for 10 min at 37°C, then centrifuged (10,000 × g, 10 min). Add 300μl of each sample to 96-well plates. Measure the absorbance at 593 nm using a microplate reader.

Malondialdehyde (MDA) assay

The relative MDA concentration in cell lysates was assessed using a lipid peroxidation assay kit (S0131S) purchased from Beyotime according to the manufacturer's instructions. Cells were seeded in 10 cm plate (5×10^6 cells per plate) and treated with corresponding drugs for 24 h. Cells were washed with ice-cold PBS and homogenized on ice in Western and IP cell lysates (P0013, Beyotime), then centrifuged (12,000 × g, 20 min) to obtain its supernatant from each homogenized sample into a microcentrifuge tube. Add 0.2ml of the assay working solution of MDA into each sample. After mixing, the mixture was heated at 100ºC or in a boiling water bath for 15 minutes. The water bath was cooled to room temperature and centrifuged at 1000g for 10 min at room temperature. 0.2ml of the supernatant was added to a 96-well plate, and absorbance was subsequently measured at 532nm using a microplate reader.

Lipid ROS assay using flow cytometer

Lipid ROS level was analyzed by flow cytometry using a reactive oxygen species assay kit (S0033S) purchased from Beyotime according to the manufacturer's instructions. DCFH-DA was diluted in serum-free medium at 1:1000 to give a final concentration of 10 micromoles/liter. The cell culture medium was removed and the appropriate volume of diluted DCFH-DA was added. The volume added was appropriate to cover the cells adequately, and usually no less than 1 ml of diluted DCFH-DA was added for one well of a six-well plate. The cells were incubated in a cell incubator at 37ºC for 20 min. Cells were washed three times with serum-free cell culture medium to adequately remove DCFH-DA that did not enter the cells. Use

ROS positive controls and corresponding drugs to stimulate cells. The intensity of fluorescence before and after stimulation was detected in real time or by time point using the 488nm excitation wavelength and 525nm emission wavelength of a flow cytometer (ACEA NovoCyte Quanteon).

Mice model

For the subcutaneous injection model, 5×10^6 FTL transfected cells, shFTL-transfected cells, and their corresponding control cells were injected subcutaneously into different sides of 4–6 week-old C57/6N or NOD/SCID mice. The tumor size was measured every 5–7 days with a caliper and calculated with the formula 0.5 × length × width × width. Mice were sacrificed 4 weeks. For the metastasis mice model, 1×10^6 FTL-transfected cells, shNRF2-transfected cells, and their corresponding control cells were injected through the hock injection of 4–6-week-old NOD/SCID mice. Mice were sacrificed around 3 months. As the NOD/SCID mice do not have any B and T cells, normal inguinal lymph nodes were very small and transparent, of which were very different from the large and solid metastatic lymph nodes. Thus, we placed only the mice with lymph node metastasis. For the subcutaneous injection model for immune function exploration, 5×10^6 FTL transfected cells and their corresponding control cells were injected subcutaneously into different sides of 4–6 week-old C57/6N mice. Mice were sacrificed on the 10th day for its tumors. The tumors were put into a 2ml EP tube containing 300-700ul of tissue digestion solution respectively, and the tissues were cut into pieces as much as possible. The tissue was bathed in water at 37℃ for 25min. During the period, the tissue block was removed and shaken every 5min for 30s. The cell suspension was transferred to a flow tube at 100w cells/ tube, and 1.5-3ml PBS was added according to the amount of red liquid, followed by centrifugation at 300g for 5min. Live/dead dye and corresponding macrophage immunophenotyping marker flow antibodies were added, and then left at room temperature for 15min and 30min. Add 1ml FACS, centrifuge at 300g for 5min, centrifuge, pour supernatant and shake off the wall hanging droplets, and finally add

200ul FACS to resuspension for flow cytometry analyses respectively.

Brusatol

The treatment of tumors via traditional Chinese medicine (TCM) is an important way to develop new drugs, and there are many studies showing the effects of Brucea javanica on tumor development, metastasis and ferroptosis[1,2]. Brusatol is a diterpenoid, a critical component extracted from the Brucea javanica plant[3], which has been shown to inhibit NRF2 activity in previous literatures[4,5]. As to the in vitro experiments, it has been reported that the viability of cancer cells was reduced in a dose-dependent manner[6]. Also, we do the dose-dependent of Brusatol in mEC2 and mEC3 cell lines (Figure 7F) for double check. As to the in vivo experiments, it has been reported Nude mice are injected with cancer cells to induce tumor growth, followed by a single injection of 2 mg/kg Brusatol. Nrf2 protein levels are significantly decreased at 24 h or 48 h postinjection[7].

Statistical analysis and reproducibility

Statistical analysis was performed, and plots were generated using GraphPad Prism 8 and IBM SPSS Statistics 25. For data with a sample size≤6, bar plots were used and all data points were shown. For data with a sample size >6, box & whiskers plots were used to visualize the data distribution. Bar plot data were presented as the mean values ± SD. Box & whiskers plots were presented as the median values ± IQR (25th and 75th percentiles) and whiskers encompass 1.5 times the IQR, as determined by the Tukey method. Violin plots were presented as kernel density estimations (KDE) annotated with median values ± IQR. For parametric data, P values were evaluated by the two-sided Student's t-test. For non-parametric data from scRNA-seq data, P values were evaluated by the two-sided Wilcoxon signed-rank test or Kruskal-Wallis one-way analysis of variance. Two-sided Pearson and Spearman correlation analysis was used to compute the correlation and statistical significance between two variables. In GSEA, both false discovery rates (FDR) and P values were used to

determine statistical significance. In survival analysis, P values were evaluated by the two-sided log-rank test.

Supplementary figure legends

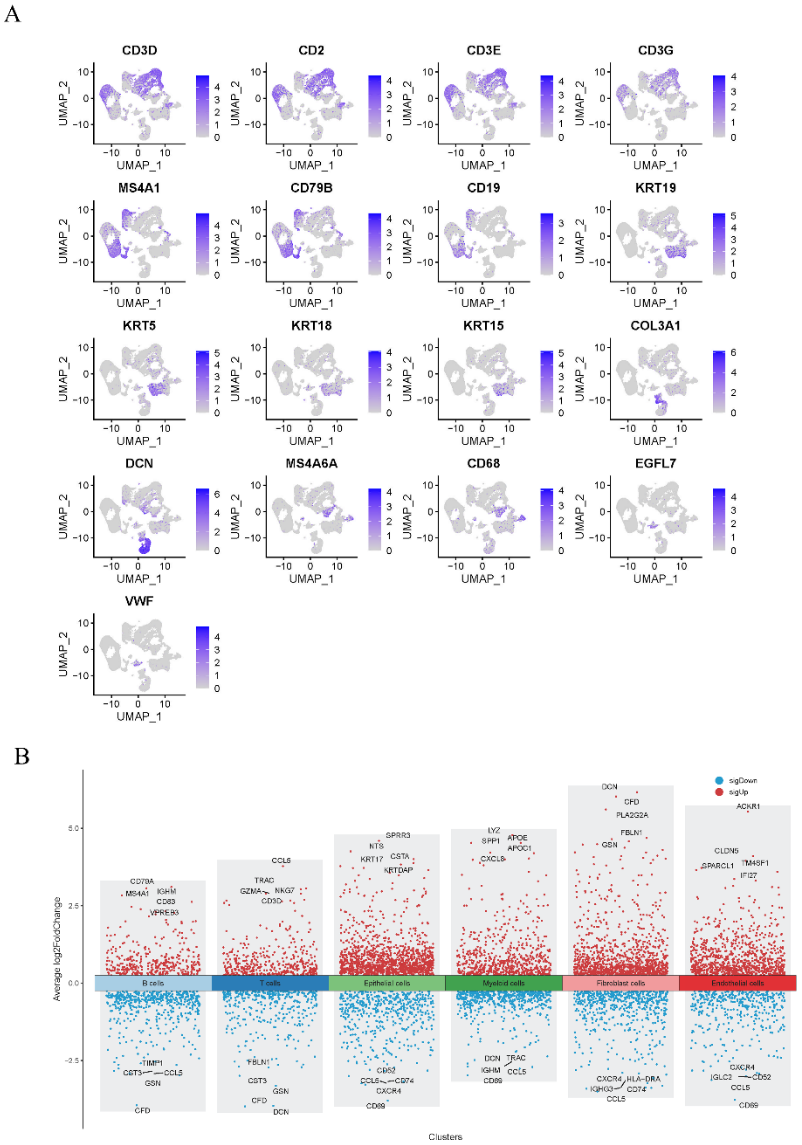

Supplementary Figure 1. Single-cell transcription atlas analyses of primary and metastatic ecosystems. (A) Umaps of specific marker genes, including B cells (MS41, CD79B, and CD19), T cells (CD3D, CD2, CD3E and CD3G), epithelial cells (KRT5, KRT15, KRT18 and KRT19), fibroblast cells (DCN and COL3A1), myeloid cells (MS4A6A, and CD68) and endothelial cells (EGFL7 and VWF). (B) Volcano

plot showing high expression genes in different cell types. The upper genes were the higher expressed group and the lower genes were the lower expressed group.

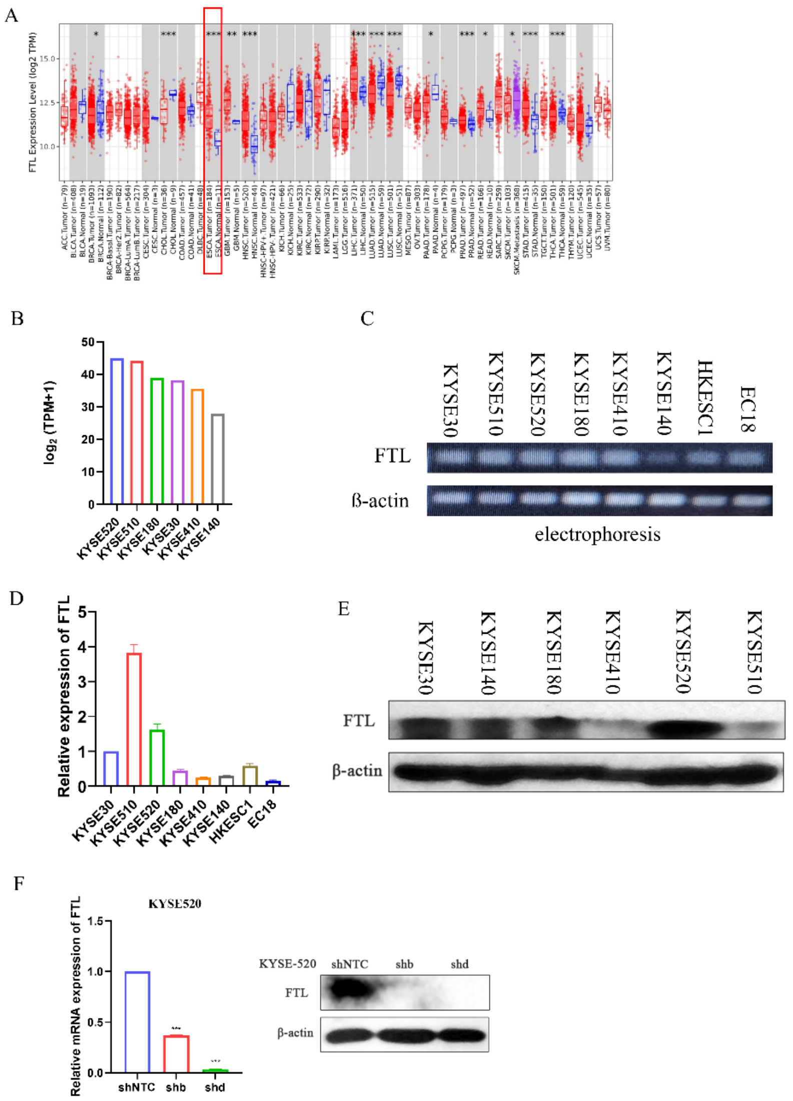

Supplementary Figure 2. FTL expression in different cell lines. (A) FTL expression levels in different tumor types via TIMER tools. (B) qPCR of FTL in different ESCC cell lines according to Cancer Cell Line Encyclopedia. (C) Electrophoresis showing FTL expression in different ESCC cell lines. (D) qPCR of FTL in different ESCC cell lines. (E) Western blotting of FTL in different ESCC cell lines. β-Actin was used as a loading control. (F) qPCR and Western blotting of FTL in KYSE520 ESCC cells transfected with lentiviruses expressing control shRNA (shNTC) and two independent shRNAs targeting FTL (shb and shd). β-Actin was

used as a loading control.

Statistical significances: *, P < 0.05; **, P < 0.01; ***, P < 0.001.

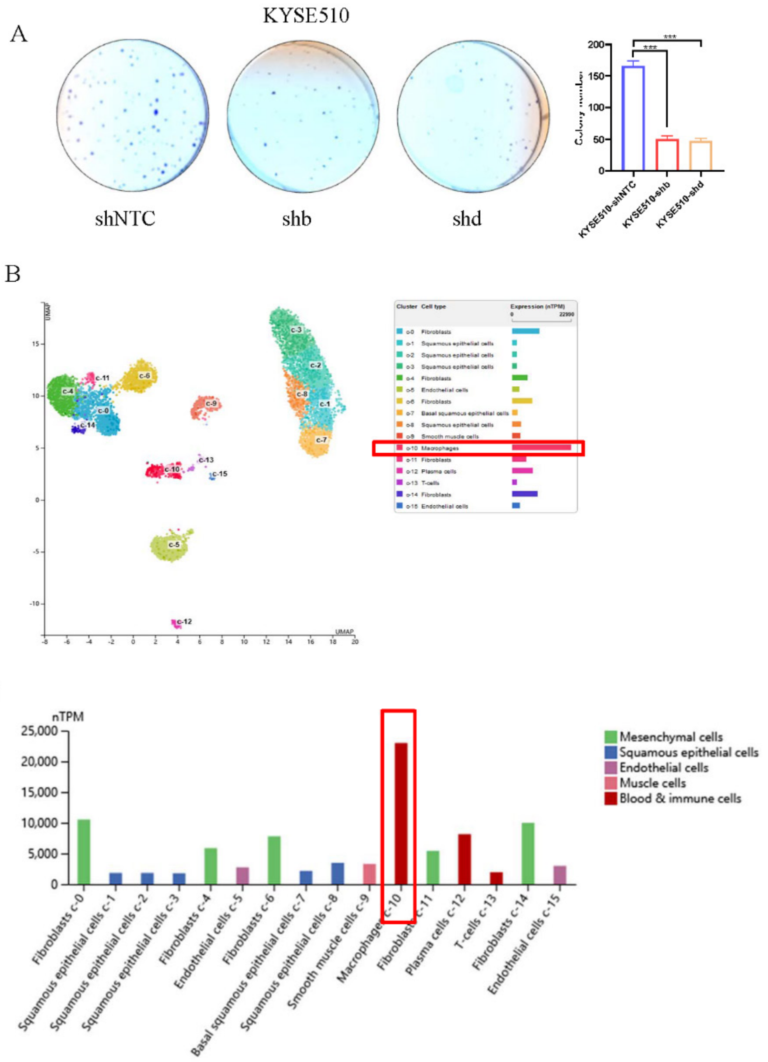

Supplementary Figure 3. FTL expression in its infiltrating different cell types.

(A) Foci formation assay showing different colony numbers in KYSE 510 cells transfected with shNTC, shb and shd. (B, C) Single cell analysis of FTL expression in different cell types in online public database.

Statistical significances: *, P < 0.05; **, P < 0.01; ***, P < 0.001.

A

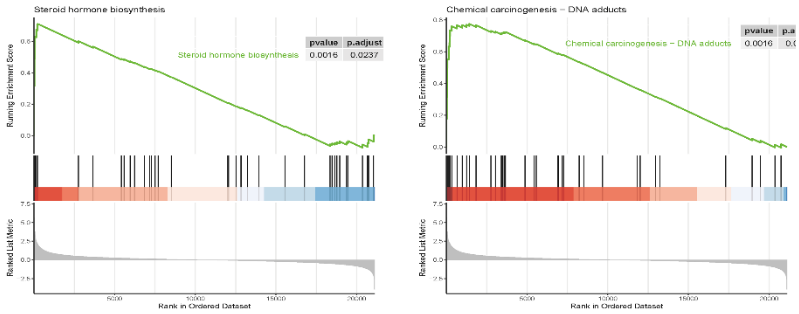

Supplementary Figure 4. Exploration of upstream and downstream genes of FTL. (A) GSEA analysis of ESCC FTLhigh and FTLlow groups in TCGA database.

Statistical significances: *, $P < 0.05$; **, $P < 0.01$; ***, $P < 0.001$.

A

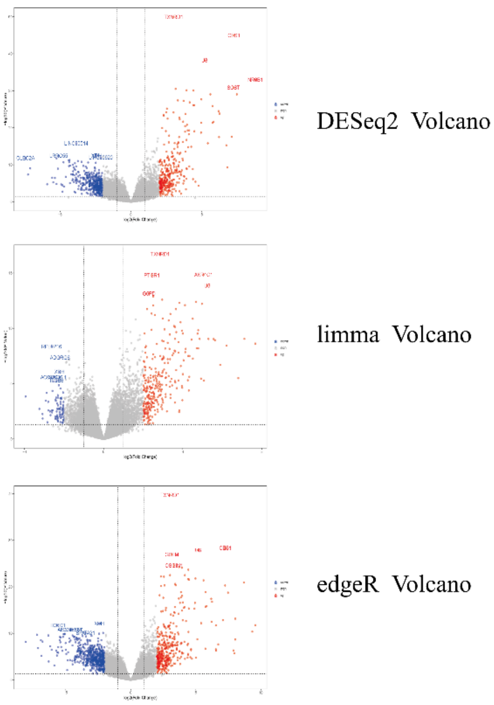

Supplementary Figure 5. Exploration of downregulated and upregulated genes of FTL in ESCC. (A) Volcano plot showing upstream and downstream genes of FTL via different algorithm.

Statistical significances: *, $P < 0.05$; **, $P < 0.01$; ***, $P < 0.001$.

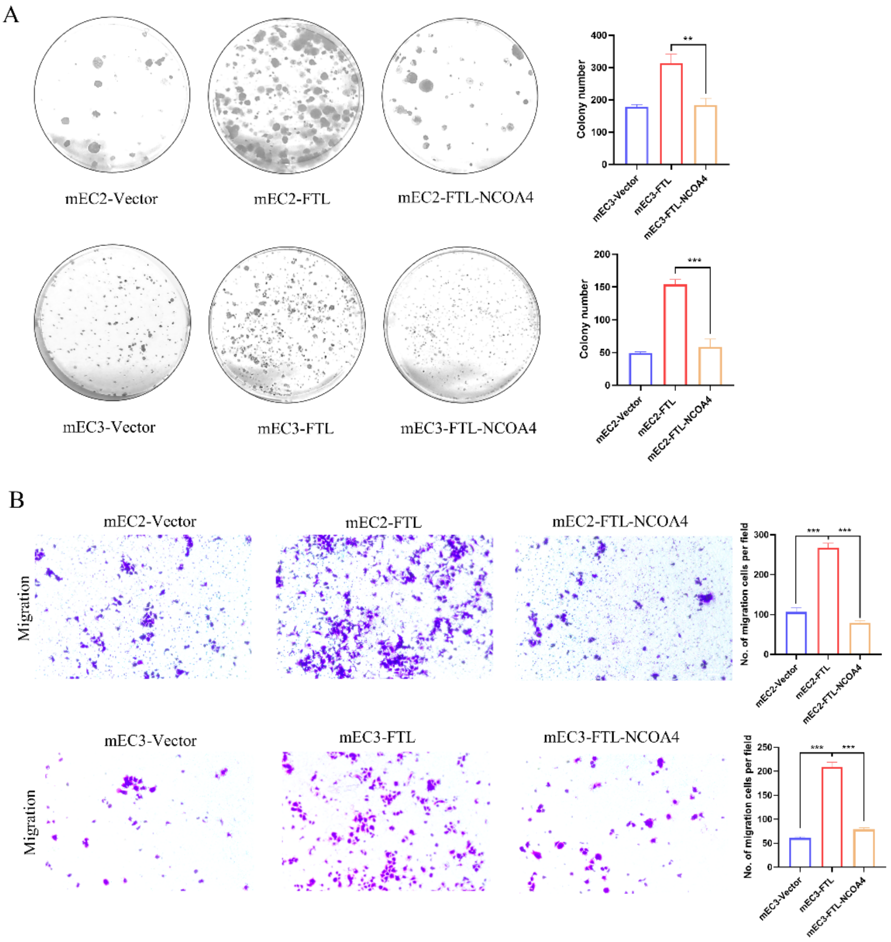

Supplementary Figure 6. Upregulation of NCOA4 impairs FTL oncogenic and metastasis effects in ESCC. (A) Foci formation assay showing different colony numbers in mEC2 and mEC3 cells transfected with vector, FTL isoform, FTL-NCOA4 isoform. (B) Representative images of transwell migration assays (left) and their summaries (right) in mEC2 and mEC3 cells transfected with vector, FTL isoform, FTL-NCOA4 isoform.

Statistical significances: *, $P < 0.05$; **, $P < 0.01$; ***, $P < 0.001$.

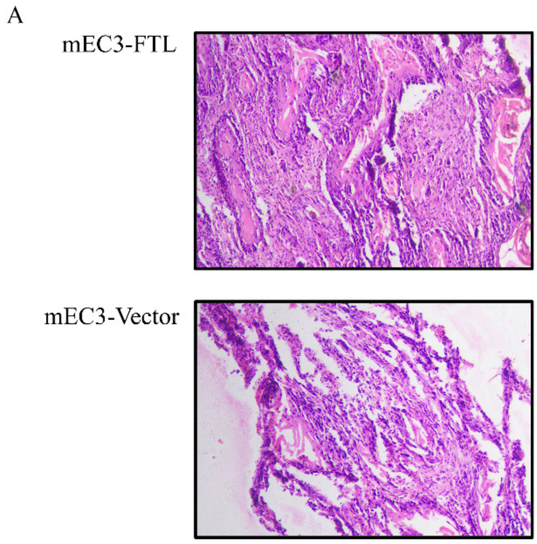

Supplementary Figure 7. H&E staining of lymph node metastases lesions in different groups. (A) Representative H&E images of the lymph node metastasis (LNM) for hock injection mice model in vector, FTL isoform groups.

Supplementary Table 1. List of primers used in this study.

Gene	Primer	Sequence
For Real-time PCR		
FTL	Forward	ATGATGTGGCTCTGGAAGGC
	Reverse	TTCATGGCGTCTGGGGTTTT
SLC40A1	Forward	TGACTGTCCTGGGCTTTGAC
	Reverse	AGACCTGTCCGAACCAAACC
GPX4	Forward	GAGGCAAGACCGAAGTAAACTAC
	Reverse	CCGAACTGGTTACACGGGAA
IREB2	Forward	TCGATGTATCTAAACTTGGCACC
	Reverse	GCCATCACAATTTCGTACAGCAG
TXNRD1	Forward	ATATGGCAAGAAGGTGATGGTCC
	Reverse	GGGCTTGTCCTAACAAAGCTG
NQO1	Forward	GAAGAGCACTGATCGTACTGGC
	Reverse	GGATACTGAAAGTTCGCAGGG
GPX2	Forward	GGTAGATTTCAATACGTTCCGGG
	Reverse	TGACAGTTCTCCTGATGTCCAAA
GSTM4	Forward	CAATGATGCAGCACTTCTCACA
	Reverse	GATCTTCTCCAAGCCCTCAAAGC
GSTM3	Forward	CGTGCGAGTCGTCTATGGTT
	Reverse	CATAGTCAGGAGCTTCCCCG
FTH1	Forward	CGCCTCCTACGTTTACCTGT
	Reverse	GAAGATTCGGCCACCTCGTT
HMOX1	Forward	AAGACTGCGTTCCTGCTCAAC
	Reverse	AAAGCCCTACAGCAACTGTCG
PRDX1	Forward	CCACGGAGATCATTGCTTTCA
	Reverse	AGGTGTATTGACCCATGCTAGAT
For clone gene		
FTL-isoform	Forward	GACACCGACTCTAGAGGATCCGCCACCATGAGCTCCCAGATTCGTCAGA
	Reverse	CGCGGGCCCTCTAGACTCGAGTTAGTCGTGCTTGAGAGTGAGCC
Knockdown-NRF2-1	Forward	CCGGCTTGAAGTCTTCAGCATGTTACTCGAGTAACATGCTGAAGACTTCAAGTTTTTG
	Reverse	AATTCAAAAACTTGAAGTCTTCAGCATGTTACTCGAGTAACATGCTGAAGACTTCAAG
Knockdown-NRF2-2	Forward	CCGGGCCTTACTCTCCCAGTGAATACTCGAGTATTCACTGGGAGAGTAAGGCTTTTTG
	Reverse	AATTCAAAAAGCCTTACTCTCCCAGTGAATACTCGAGTATTCACTGGGAGAGTAAGGC

Supplementary Table 2. List of antibodies used in this study.

Antibody	Size (kDa)	Vendor	Cat No.	Application
Rabbit anti-FTL	10-20	ABclonal	A1768	WB, 1: 1000; IHC, 1:100
Mouse anti-FTL	10-20	Novus	H00002512-M16	IF, 1: 100; IP, 1 μg/mg
Rabbit anti-NRF2	95-140	Novus	NBP1-32822	IF, 1:500; WB, 1:1000
Rabbit anti-KEAP1	60-75	ABclonal	A1820	WB, 1:1000
Rabbit anti-HO-1	25-35	ABclonal	A11102	WB, 1:1000
Rabbit anti-SLC7A11	50-70	ABclonal	A2413	WB, 1:1000
Rabbit anti-IREB2	100-150	ABclonal	A6382	WB, 1:1000
Rabbit anti-FTH1	20	ABclonal	A19544	WB, 1:1000
Mouse anti-NCOA4	50-75	Sigma-Aldrich	SAB1409837	IF, 1:100; WB, 1:1000
Rabbit anti-NCOA4	71-117	Abcam	ab86707	IP, 1 μg/mg; WB, 1:1000
Mouse anti-E-cadherin	100-140	Abcam	ab76055	WB, 1:1000
Rabbit anti-β-catenin	80-100	Cell signaling	CST8480	WB, 1:1000
Rabbit anti-Vimentin	50-60	Cell signaling	CST5741s	WB, 1:1000
Rabbit anti-Snail	30	Cell signaling	CST3879s	WB, 1:1000
Rabbit anti-Slug	30	Cell signaling	C19G7	WB, 1:1000
Mouse anti-β-actin	43	Abcam	ab6276	WB, 1:5000

[1] He T, Zhou F, Su A, Zhang Y, Xing Z, Mi L, Li Z, Wu W. Brusatol: A potential sensitizing agent for cancer therapy from Brucea javanica. Biomed Pharmacother. 2023 Feb;158: 114134. doi: 10.1016/j.biopha.2022.114134.

[2] Xing S, Nong F, Wang Y, Huang D, Qin J, Chen YF, He DH, Wu PE, Huang H, Zhan R, Xu H, Liu YQ. Brusatol has therapeutic efficacy in non-small cell lung cancer by targeting Skp1 to inhibit cancer growth and metastasis. Pharmacol Res. 2022 Feb;176: 106059. doi: 10.1016/j.phrs.2022.106059.

[3] Lee KH, Okano M, Hall IH, Brent DA, Soltmann B. Antitumor agents XLV: Bisbrusatolyl and brusatolyl esters and related compounds as novel potent antileukemic agents. J Pharm Sci. 1982 Mar;71(3):338-45. doi: 10.1002/jps.2600710320.

[4] Park JE, Leem YH, Park JS, Kim SE, Kim HS. Astrocytic Nrf2 Mediates the Neuroprotective and Anti-Inflammatory Effects of Nootkatone in an MPTP-Induced Parkinson's Disease Mouse Model. Antioxidants (Basel). 2023 Nov 13;12(11):1999. doi: 10.3390/antiox12111999.

[5] Yan J, Li Z, Liang Y, Yang C, Ou W, Mo H, Tang M, Chen D, Zhong C, Que D, Feng L, Xiao H, Song X, Yang P. Fucoxanthin alleviated myocardial ischemia and reperfusion injury through inhibition of ferroptosis via the NRF2 signaling pathway. Food Funct. 2023 Nov 13;14(22):10052-10068. doi: 10.1039/d3fo02633g.

[6] Chen HM, Lai ZQ, Liao HJ, Xie JH, Xian YF, Chen YL, Ip SP, Lin ZX, Su ZR. Synergistic antitumor effect of brusatol combined with cisplatin on colorectal cancer cells. Int J Mol Med. 2018 Mar;41(3):1447-1454. doi: 10.3892/ijmm.2018.3372.

[7] Ren D, Villeneuve NF, Jiang T, Wu T, Lau A, Toppin HA, Zhang DD. Brusatol enhances the efficacy of chemotherapy by inhibiting the Nrf2-mediated defense mechanism. Proc Natl Acad Sci U S A. 2011 Jan 25;108(4):1433-8. doi: 10.1073/pnas.1014275108.
